# Release of P-TEFb from the Super Elongation Complex promotes HIV-1 latency reversal

**DOI:** 10.1371/journal.ppat.1012083

**Published:** 2024-09-11

**Authors:** William J. Cisneros, Shimaa H. A. Soliman, Miriam Walter, Lacy M. Simons, Daphne Cornish, Simone De Fabritiis, Ariel W. Halle, Eun-Young Kim, Steven M. Wolinsky, Ramon Lorenzo-Redondo, Ali Shilatifard, Judd F. Hultquist

**Affiliations:** 1 Division of Infectious Diseases, Department of Medicine, Feinberg School of Medicine, Northwestern University, Chicago, Illinois, United States of America; 2 Center for Pathogen Genomics and Microbial Evolution, Havey Institute for Global Health, Feinberg School of Medicine, Northwestern University, Chicago, Illinois, United States of America; 3 Simpson Querrey Institute for Epigenetics, Department of Biochemistry and Molecular Genetics Feinberg School of Medicine, Northwestern University, Chicago, Illinois, United States of America; Emory University, UNITED STATES OF AMERICA

## Abstract

The persistence of HIV-1 in long-lived latent reservoirs during suppressive antiretroviral therapy (ART) remains one of the principal barriers to a functional cure. Blocks to transcriptional elongation play a central role in maintaining the latent state, and several latency reversal strategies focus on the release of positive transcription elongation factor b (P-TEFb) from sequestration by negative regulatory complexes, such as the 7SK complex and BRD4. Another major cellular reservoir of P-TEFb is in Super Elongation Complexes (SECs), which play broad regulatory roles in host gene expression. Still, it is unknown if the release of P-TEFb from SECs is a viable latency reversal strategy. Here, we demonstrate that the SEC is not required for HIV-1 replication in primary CD4+ T cells and that a small molecular inhibitor of the P-TEFb/SEC interaction (termed KL-2) increases viral transcription. KL-2 acts synergistically with other latency reversing agents (LRAs) to reactivate viral transcription in several cell line models of latency in a manner that is, at least in part, dependent on the viral Tat protein. Finally, we demonstrate that KL-2 enhances viral reactivation in peripheral blood mononuclear cells (PBMCs) from people living with HIV (PLWH) on suppressive ART, most notably in combination with inhibitor of apoptosis protein antagonists (IAPi). Taken together, these results suggest that the release of P-TEFb from cellular SECs may be a novel route for HIV-1 latency reactivation.

## Author summary

Since the start of the HIV pandemic, it is estimated that nearly 86 million people have been infected with the virus, and about 40 million people have died. Modern antiretroviral therapies potently restrict viral replication and prevent the onset of AIDS, saving millions of lives. However, these therapies are not curative due to the persistence of the virus in a silenced or ‘latent’ state in long-lived cells of the body. One proposed strategy to clear this latent reservoir, termed “shock and kill”, is to activate these silenced viruses such that the infected cells can be cleared from the body by the immune system. While several drugs have been developed that can activate latent viruses, none have proven effective at reducing the size of the latent reservoir in patients in clinical trials. Here, we describe a new method for latency reactivation using a small molecule inhibitor of a human protein complex called the Super Elongation Complex (SEC). Inhibiting the SEC enhances viral transcription during active infection and triggers the reactivation of latent viruses, especially when in combination with other latency reversing agents. These results pave the way for developing more effective strategies to reactivate latent viruses towards a functional cure.

## Introduction

The persistence of replication-competent, but transcriptionally inhibited HIV-1 proviral DNA in long-lived, latent cellular reservoirs is a significant barrier to the development of a functional cure [1,2] Even after long-term, suppressive antiretroviral therapy (ART), spontaneous reactivation of proviral gene expression from the latent reservoir is sufficient to initiate viral rebound shortly after ART cessation, thus requiring life-long adherence [3–5]. Multifaceted and heterogenous blocks to viral gene expression establish and maintain HIV-1 latency at the epigenetic, transcriptional, and post-transcriptional levels [2]. Several strategies to either reactivate latent proviruses and clear infected cells (*i*.*e*., “shock and kill”) or reinforce latency to prevent spontaneous reactivation (*i*.*e*., “block and lock”) are currently under investigation [6,7].

While many small molecule latency reversing agents (LRAs) have been described that reactivate latent proviruses *ex vivo*, they have had little success in clinical trials [8,9]. This failure is partly due to the latent reservoir’s heterogenous nature, such that treatment by a single agent that acts to target a specific block may only ever reactivate a small fraction of proviruses *in vivo* [10]. Even if transcriptional reactivation is achieved, it is unlikely this is sufficient to result in the clearance of these infected cells without additional immune augmentation such as the administration of antibodies, vaccines, or immunotherapies to prime the immune system [6]. Given these limitations, much research is now focused on combinatorial approaches to trigger more widespread and robust reactivation [11]. For example, a recent study reported synergistic reactivation potential between an activator of non-canonical NF-kB signaling (AZD5582) and BET bromodomain inhibitors that act to lift blocks to transcriptional initiation and elongation, respectively [12].

Blocks to transcriptional elongation are major contributors to establishing and maintaining HIV-1 latency [13,14]. After integration of the proviral DNA, RNA Polymerase II (RNA Pol II) is recruited to the transcription start site by transcription factors that bind *cis*-elements in the HIV-1 promoter region. After transcriptional initiation, RNA Pol II synthesizes 20–60 nucleotides before stalling through a well-conserved process known as promoter-proximal pausing [15]. Pausing is enforced by several negative elongation factors, including negative elongation factor (NELF), DRB Sensitivity Inducing Factor (DSIF), and the RNA Polymerase II Associated Factor 1 (PAF1) complex [16–18]. Pause release is regulated by positive transcription elongation factor-b (P-TEFb), a heterodimeric protein complex composed of cyclin-dependent kinase 9 (CDK9) and cyclin T1 (CCNT1) [19–21]. P-TEFb phosphorylates the C-terminal tail of RNA Pol II and several negative elongation factors, which collectively license transcriptional elongation [22–24].

Recruitment of P-TEFb to sites of nascent transcription is a highly regulated process mediated by several cellular complexes. The majority of cellular P-TEFb is sequestered in an inactive state by the 7SK ribonucleoprotein (RNP) complex [25,26]. Diverse extracellular stimuli and intracellular signals can induce the release of P-TEFb from the 7SK complex [27–29] where it can be recruited to sites of nascent transcription by transcription factors (*i*.*e*., NF-kB and c-MYC) [30–32], epigenetic regulators (*i*.*e*., BRD4) [33,34], or super elongation complexes (SECs) composed of an ARF4/FMR2 (AFF) family scaffold protein in complex with AF9, ENL, an eleven-nineteen Lys-rich leukemia (ELL) family protein, and an ELL-associated factor (EAF) protein [35]. To circumvent this regulatory step, HIV-1 encodes a trans-activator protein (Tat) that binds to and recruits P-TEFb specifically to sites of nascent proviral transcription through recognition of a transactivation response (TAR) RNA stem loop produced at the immediate 3’ end of all viral RNA transcripts [36–38].

The distribution of and competition for P-TEFb binding among different complexes is an area of active investigation, with several strategies to enhance the biogenesis or availability of P-TEFb showing promise for HIV-1 latency reversal. For example, BET bromodomain inhibitors (such as JQ1) have been shown to be potent LRAs in *ex vivo* models by releasing P-TEFb from BRD4 [12]. Likewise, the release of P-TEFb from the 7SK RNP complex has been shown to reactivate latent proviruses in *ex vivo* models [39]. That being said, the release of P-TEFb from the 7SK RNP complex has been shown to directly correlate with increased BRD4 binding, suggesting that release from any one complex will not necessarily increase the amount of unbound P-TEFb or the amount recruited to specific sites of transcription [33]. Furthermore, post-translational modification of P-TEFb (*i*.*e*., through phosphorylation of CDK9 at Serine 175) has been shown to influence P-TEFb distribution in certain regulatory complexes, again highlighting the unique properties of release from each complex [40].

While the release of P-TEFb from the 7SK RNP complex and BET proteins such as BRD4 have been explored as strategies for HIV-1 latency reversal, the release of P-TEFb from SECs has not been explored. Previous work has demonstrated that HIV-1 Tat biochemically co-purifies with several SEC proteins [41,42], though the reason for this is unclear as they seemingly have functionally redundant purposes in P-TEFb recruitment. In this study, we test the hypothesis that the SEC is not necessary for HIV-1 viral transcription and that the release of P-TEFb from cellular SEC complexes can serve as a novel strategy to reactivate latent HIV-1 proviruses.

## Results

### The Super Elongation Complex is not required for HIV-1 replication in primary CD4+ T cells

**Fig 1:**
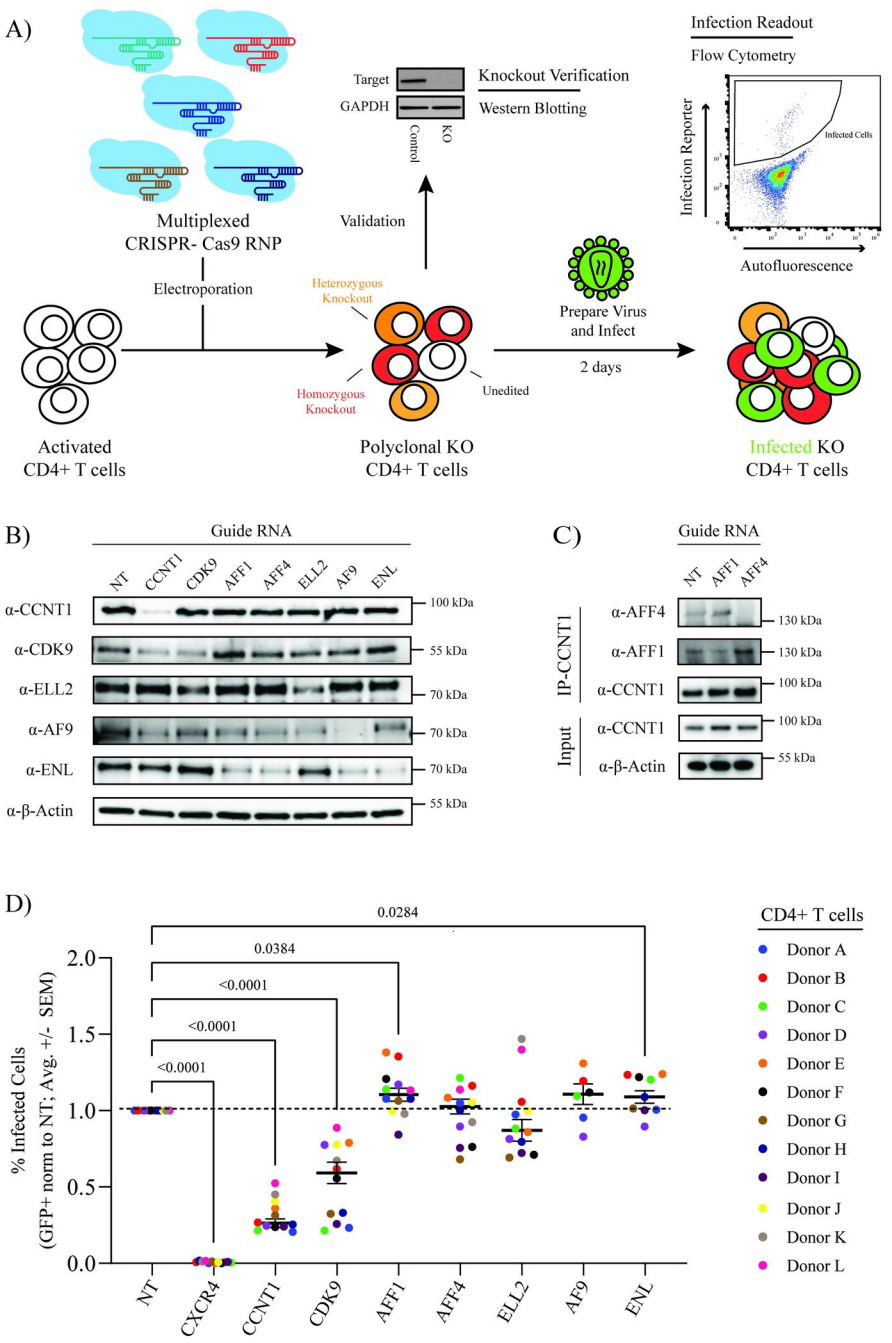
Knock-out of Super Elongation Complex members does not decrease HIV-1 infection in primary CD4+ T cells. **A)** Experimental schematic of multiplex CRISPR-Cas9 gene editing of primary CD4+ T cells. **B)** Immunoblots to assess CCNT1, CDK9, ELL2, AF9, and ENL knock-out efficiency in primary CD4+ T cells relative to a non-targeting (NT) control (1 representative donor). **C)** Immunoblots to assess AFF1 and AFF4 knock-out efficiency in primary CD4+ T cells relative to a NT control following immunoprecipitation of CCNT1 (1 representative donor). **D)** Percent infected (GFP+) primary CD4+ T cells (normalized to the donor-matched NT control) 48 hours after challenge with HIV-1 NL4.3 Nef:IRES:GFP. Each dot represents the average of technical triplicates; the black line represents the mean of means ± standard error of means. n = 12 donors for NT, CXCR4, CCNT1, CDK9, AFF1, AFF4, and ELL2; n = 6 donors for AF9; and n = 9 donors for ENL. Statistics were calculated by two-way ANOVA with Dunnet’s Multiple Comparison Test; significant p-values (p < 0.05) are shown.

While biochemical purification of HIV-1 Tat from cell lines has been shown to pull down other SEC members besides P-TEFb (including AFF1, AFF4, ELL2, ENL and AF9), the role of the SEC in HIV-1 replication in primary CD4+ T cells is unclear. To determine whether the SEC is required for HIV-1 replication in primary CD4+ T cells, we used multiplexed CRISPR-Cas9 RNPs to knock-out expression of each SEC component in cells from multiple healthy donors (Fig 1A) [43–45]. Each multiplexed reaction consisted of 4 or 5 independent guide RNA targeting the same gene [45]. A non-targeting (NT) guide RNA was used as a negative control whereas a previously validated guide RNA targeting the HIV-1 co-receptor gene, *CXCR4*, was used as a positive control [43]. Immunoblots of protein lysates collected 72 hours after CRISPR-Cas9 RNP electroporation demonstrated depletion of each target (Fig 1B). Visualization of AFF1 and AFF4 depletion required CCNT1 immunoprecipitation, likely due to their low steady-state levels in CD4+ T cells and low-affinity antibodies (Fig 1C). Notably, the depletion of some targets had secondary effects on other complex members. For example, knock-out of *CCNT1* decreased CDK9 steady state levels and knock-out of *AFF1*, *AFF4*, and *AF9* each decreased ENL steady-state levels. None of the knockouts were found to decrease cell viability significantly at this time point (S1A and S1B Fig).

To determine the impact of SEC component knock-out on HIV-1 replication, we challenged each cell population with replication-competent HIV-1 NL4.3 containing an IRES-driven GFP reporter inserted after Nef (HIV-1 NL4.3 Nef:IRES:GFP) in technical triplicate. The percentage of infected cells was quantified at two-days post-challenge by flow cytometry (S1C Fig) and normalized to the NT control (Fig 1D). Knock-out of *CXCR4* ablated CXCR4-tropic NL4.3 strain infection as expected. Knock-out of each P-TEFb component (*CCNT1*, *CDK9*) significantly decreased infection. However, knock-out of the other SEC components either did not alter HIV-1 infection (*AFF4*, *ELL2*, *AF9*) or resulted in a very slight, but statistically significant increase in infection (*AFF1*, *ENL*). These data suggest that, while P-TEFb is required, the rest of the SEC is dispensable for HIV-1 replication in primary CD4+ T cells.

### The SEC inhibitor KL-2 enhances HIV-1 infection in primary CD4+ T cells

**Fig 2:**
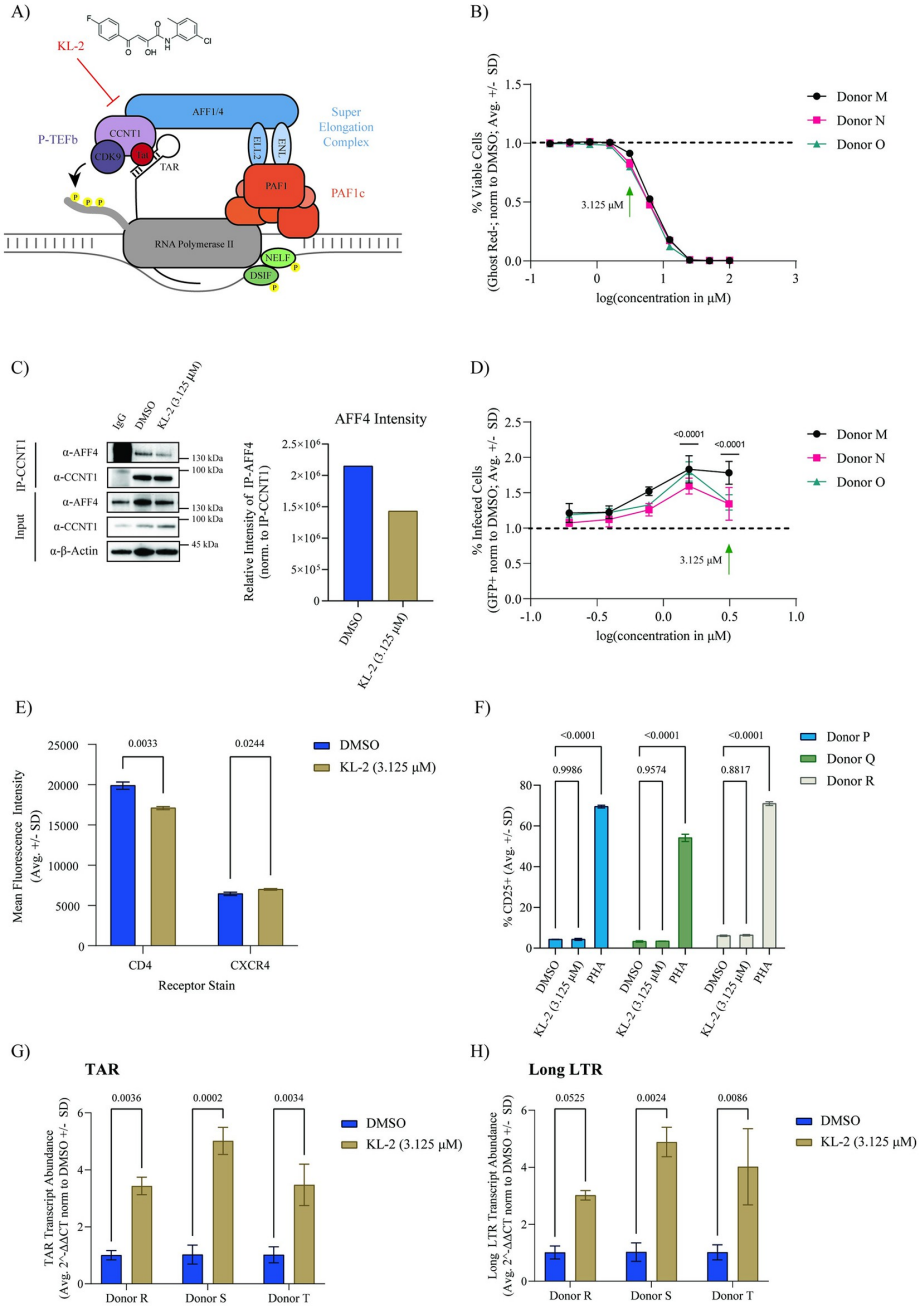
Super Elongation Complex disruptor KL-2 increases HIV-1 infection in primary CD4+ T cells. **A)** Model of Tat-mediated recruitment of P-TEFb to paused RNA Pol II at sites of nascent HIV-1 proviral transcription. **B)** Percent viable primary human CD4+ T cells (normalized to the donor-matched DMSO control) after 72 hours of treatment with increasing concentrations of KL-2 as measured by amine dye staining and flow cytometry. Data represent the average ± standard deviation of technical triplicates; the green arrow indicates the concentration chosen for downstream experiments in primary CD4+ T cells. **C)** Immunoblots for AFF4 and CCNT1 following CCNT1 immunoprecipitation from primary CD4+ T cells treated for 24-hours with either DMSO or 3.125 μM KL-2 (1 representative donor). Quantification of immunoprecipitated AFF4 levels normalized to immunoprecipitated CCNT1 is shown on the right. **D)** Percent infected (GFP+) primary CD4+ T cells (normalized to the donor-matched DMSO control) 48 hours after challenge with HIV-1 NL4.3 Nef:IRES:GFP in the presence of increasing concentrations of KL-2 (24 hours pre-treatment before challenge). Data represent the average ± standard deviation of technical triplicates (n = 3 donors); statistics were calculated relative to the DMSO control by two-way ANOVA and Sidak’s Multiple Comparison test with significant p-values (p < 0.05) shown. **E)** Percent of activated (CD25+) primary CD4+ T cells following treatment with DMSO, 3.125 μM KL-2, or 1μg/mL PHA for 48 hours as measured by immunostaining and flow cytometry. Data represent the average ± standard deviation of technical triplicates (n = 3 donors); statistics were calculated by two-way ANOVA and Sidak’s Multiple Comparison test with p-values shown. **F)** Mean fluorescence intensity (MFI) of CD4 and CXCR4 on primary CD4+ T cells following treatment with DMSO or 3.125 μM KL-2 for 48 hours as measured by immunostaining and flow cytometry. Data represent the average ± standard deviation of technical triplicates (n = 1 donor); statistics were calculated by Student’s t-test with p-values shown. **G)** Relative transcript levels of HIV-1 TAR and **H)** long LTR to human *β-Actin* in activated primary CD4+ T cells after 48-hours of challenge with HIV-1 in the presence or absence of a 24-hour pretreatment with KL-2. Data represent the mean of means of 3 biological replicates in technical duplicate ± standard error; statistics were calculated by two-way ANOVA with Sidak’s Multiple Comparison test.

Seeing that knock-out of several SEC components had no effect on HIV-1 replication in primary CD4+ T cells, we next wanted to determine the impact of chemical perturbation of this complex using the previously described SEC inhibitor, KL-2 [32,46]. KL-2 inhibits SEC function by disrupting the interaction between CCNT1 and AFF1/AFF4 without altering overall protein levels of P-TEFb (Fig 2A) [32]. Given that the SEC is dispensable for HIV-1 replication, we hypothesized that KL-2 treatment may increase the availability of P-TEFb for recruitment to sites of viral transcription. Activated CD4+ T cells from three independent donors were treated over a range of KL-2 concentrations for 24 hours and then challenged with HIV-1 NL4.3 Nef:IRES:GFP for 48 hours in technical triplicate. Higher concentrations of KL-2 dramatically decreased cell viability, with 3.125 μM being the highest tolerated dose with minimal toxicity (Fig 2B). This dose was sufficient to inhibit the CCNT1:AFF4 interaction in primary CD4+ T cells within 24 hours as determined by CCNT1 immunoprecipitation (Fig 2C). Compared to DMSO-treated control cells, KL-2 treatment resulted in a dose-dependent increase in infection with significant increases observed at 1.56 μM and 3.125 μM (Fig 2D).

Given that receptor and co-receptor expression can alter HIV-1 susceptibility, we next tested the impact of KL-2 treatment on CD4 and CXCR4 cell surface expression. Activated, primary CD4+ T cells from one representative donor were treated with 3.125 μM KL-2 or DMSO for 48 hours prior to immunostaining and flow cytometry (S2A and S2B Fig). KL-2 treatment resulted in a slight, but significant increase in CXCR4 expression (mean fluorescence intensity) and a slight, but significant decrease in CD4 expression (Fig 2E). To determine if KL-2 could alternately impact CD4+ T cell activation, we treated unstimulated CD4+ T cells from three independent donors with DMSO, 3.125 μM KL-2, or the T cell mitogen Phytohemagglutinin (PHA) for 48 hours. Unlike PHA, which resulted in robust activation, KL-2 treatment did not impact T cell activation as measured by CD25 cell surface staining (Fig 2F). These results suggest that increased infection in the presence of KL-2 is likely not driven by changes in receptor expression.

To assess whether this increase in infection was due to enhanced viral transcription, activated CD4+ T cells from three healthy donors were treated with 3.125 μM KL-2 or DMSO for 24 hours and then challenged with HIV-1 NL4.3 Nef:IRES:GFP in technical triplicate. After 48 hours, RNA from the infected cultures was isolated and the expression of viral transcripts was measured using quantitative reverse transcription PCR (qRT-PCR). Quantification of TAR and long LTR transcripts were used to measure viral transcriptional initiation and elongation, respectively, relative to the human housekeeping gene, *β-Actin*. We found that KL-2 treatment significantly increased the expression of both TAR and long LTR transcripts (Fig 2G and 2H).

To determine if KL-2 has similar effects on HIV-1 replication across different cell types, CHME microglial cells engineered to express CXCR4 [47] (CHME-4X4 cells), were pretreated with KL-2 over a range of concentrations for 24 hours then challenged with replication-competent HIV-1 NL4.3 containing a nano-luciferase reporter in place of Nef (Nef:Nano-Luc). As in the primary CD4+ T cells, we observed a dose-dependent increase in infection, though higher concentrations again resulted in viability defects (S2C and S2D Fig). Taken together, these data support the genetic data above that the interaction between P-TEFb and the larger SEC is not required for HIV-1 replication in primary CD4+ T cells and that SEC disruption can enhance viral transcription.

### KL-2 synergizes with other latency reversing agents to reactivate latent infection in J-Lat models

**Fig 3:**
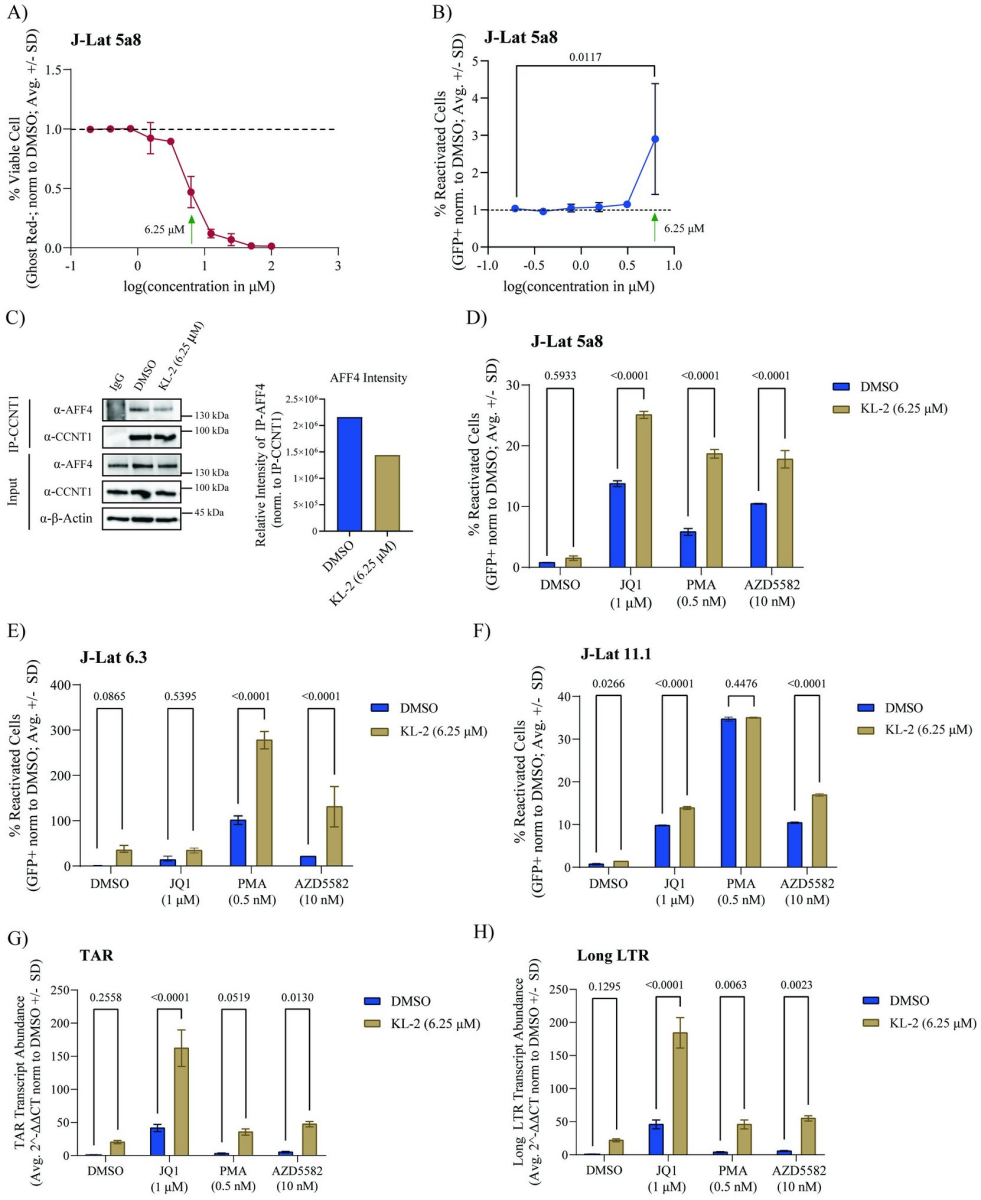
KL-2 enhances latency reversing agent activity in J-Lat cell lines. **A)** Percent viable J-Lat 5A8 cells (normalized to the DMSO control) after 48 hours of treatment with increasing concentrations of KL-2 as measured by amine dye staining and flow cytometry. Data represent the average ± standard deviation of technical triplicates; the green arrow indicates the concentration chosen for downstream experiments in cell lines. **B)** Percent reactivated (GFP+) J-Lat 5A8 cells (normalized to the DMSO control) after 48 hours of treatment with increasing concentrations of KL-2. Data represent the average ± standard deviation of technical triplicates; statistics were calculated relative to the DMSO control by two-way ANOVA and Sidak’s Multiple Comparison test. **C)** Immunoblots for AFF4 and CCNT1 following CCNT1 immunoprecipitation from J-Lat 5A8 cells treated for 24 hours with either DMSO or 6.25 μM KL-2. Quantification of immunoprecipitated AFF4 levels normalized to immunoprecipitated CCNT1 is shown on the right. **D)** Percent reactivated (GFP+) J-Lat 5A8 cells, **E)** J-Lat 6.3 cells, or **F)** J-Lat 11.1 cells (normalized to the DMSO control) after 48 hours of treatment with the indicated compounds in the presence or absence of KL-2. Data represent the average ± standard deviation of technical triplicates; statistics were calculated by two-way ANOVA with Sidak’s Multiple Comparison test. Relative transcript levels of HIV-1 TAR **(G)** and long LTR **(H)** to human *β-Actin* in J-Lat 5A8 cells after 48 hours of treatment with the indicated compounds in the presence or absence of KL-2 (normalized to the DMSO control). Data represent the mean of means of 3 biological replicates in technical duplicate ± standard error; statistics were calculated by two-way ANOVA with Sidak’s Multiple Comparison test.

The release of P-TEFb from sequestration by BRD4 or the 7SK complex has proven effective in latency reactivation [39,48]. Given that the SEC is not required for HIV-1 replication, we hypothesized that releasing P-TEFb from cellular SECs may also reactivate latent proviruses. To test this hypothesis, we treated J-Lat 5a8 cells (a Jurkat subclone harboring a silent, integrated non-replicative full-length provirus and a GFP reporter gene [49]) over a range of KL-2 concentrations for 48 hours. As in primary CD4+ T cells, higher concentrations of KL-2 decreased J-Lat 5A8 cell viability (Fig 3A). However, treatment with KL-2 alone did not significantly increase proviral reactivation (GFP+ cells as measured by flow cytometry) until 6.25 μM, at which dose the cells are only about 50% viable (Fig 3A and 3B). This dose was sufficient to inhibit the CCNT1:AFF4 interaction in J-Lat 5A8 cells within 24 hours, as determined by CCNT1 immunoprecipitation (Fig 3C).

While KL-2 was not sufficient to reactivate latent proviruses in J-Lat 5A8 cells, we hypothesized that it could enhance the activity of other latency reversing agents (LRAs) that act through different mechanisms, similar to the PAF1 complex inhibitors we reported previously [16]. To test this hypothesis, we treated J-Lat 5a8 cells with several well-characterized LRAs in the presence or absence of KL-2, including JQ1 (a BRD4 inhibitor that enhances P-TEFb availability and relieves chromosomal repression), Phorbol 12-myristate 13-acetate (PMA, a protein kinase C activator that activates canonical NF-kB transcription), and AZD5582 (an IAP antagonist that activates non-canonical NF-kB transcription). While KL-2 alone did not significantly increase reactivation compared to the DMSO control, it significantly enhanced reactivation in the presence of the other three LRAs (Fig 3D). A similar pattern was observed in two other J-Lat clones with different proviral integration sites: J-Lat 6.3 cells (Fig 3E) and J-Lat 11.1 cells (Fig 3F). Paired cell viability data is provided in S3A–S3C Fig.

To assess whether the effect of KL-2 in combination with other LRAs was synergistic, we calculated excess over Bliss scores [50] for KL-2 in combination with JQ1 and AZD5582. Excess over Bliss calculations measure whether the observed combinatorial effects at given concentrations are above or below predicted additivity, where a score of 0 is additive, scores above 0 are considered synergistic, and scores less than 0 are considered antagonistic. At the fixed 6.25 μM concentration of KL-2 used in the previous assays in combination with ascending doses of AZD5582, we observed positive excess over Bliss scores ranging from 0.46 to 0.94, suggestive of synergistic effects (S3D Fig; top). Notably, at a fixed concentration of AZD5582 (10 nM), we observe strictly additive effects of KL-2 until the effective concentration of 6.25 μM is reached. Similar trends are observed with JQ1. At the fixed 6.25 μM concentration of KL-2 with ascending doses of JQ1, we again saw positive excess over Bliss scores ranging from 0.54 to 0.94 (S3D Fig; bottom). Likewise, at a fixed concentration of JQ1 (1 μM), we observe strictly additive effects of KL-2 until the effective concentration of 6.25 μM is reached. This data would indicate that at concentrations of KL-2 lower than 6.25 μM we are likely not seeing optimal disruption of the SEC, but upon disruption at the effective dose of KL-2, the release of P-TEFb acts synergistically with the LRAs tested.

Given the role of KL-2 in P-TEFb release from the SEC, we hypothesized that treatment with KL-2 would enhance transcriptional elongation. To test this, we repeated our combinatorial LRA treatments with and without KL-2 in J-Lat 5A8 cells and extracted RNA at 48 hours post-treatment to quantify viral transcripts using qRT-PCR. HIV-1 long LTR transcripts, made after the start of transcriptional elongation, were not statistically increased upon KL-2 treatment alone, but were enhanced by KL-2 addition to every other tested LRA (Fig 3H). HIV-1 TAR transcripts, made immediately after transcriptional initiation, were not statistically increased upon KL-2 addition either alone or in the presence of PMA, however, KL-2 treatment did significantly increase transcriptional initiation in the presence of JQ1 and AZD5582 (Fig 3G). Taken together, these results suggest that KL-2 can enhance the latency reactivation activity of other LRAs in J-Lat models by increasing the availability of P-TEFb and promoting transcriptional elongation. However, additional impacts on transcriptional initiation cannot be ruled out as also observed in our primary CD4+ T cell data (Fig 2G).

### KL-2 treatment promotes RNA Pol II and P-TEFb occupancy across the proviral genome

**Fig 4:**
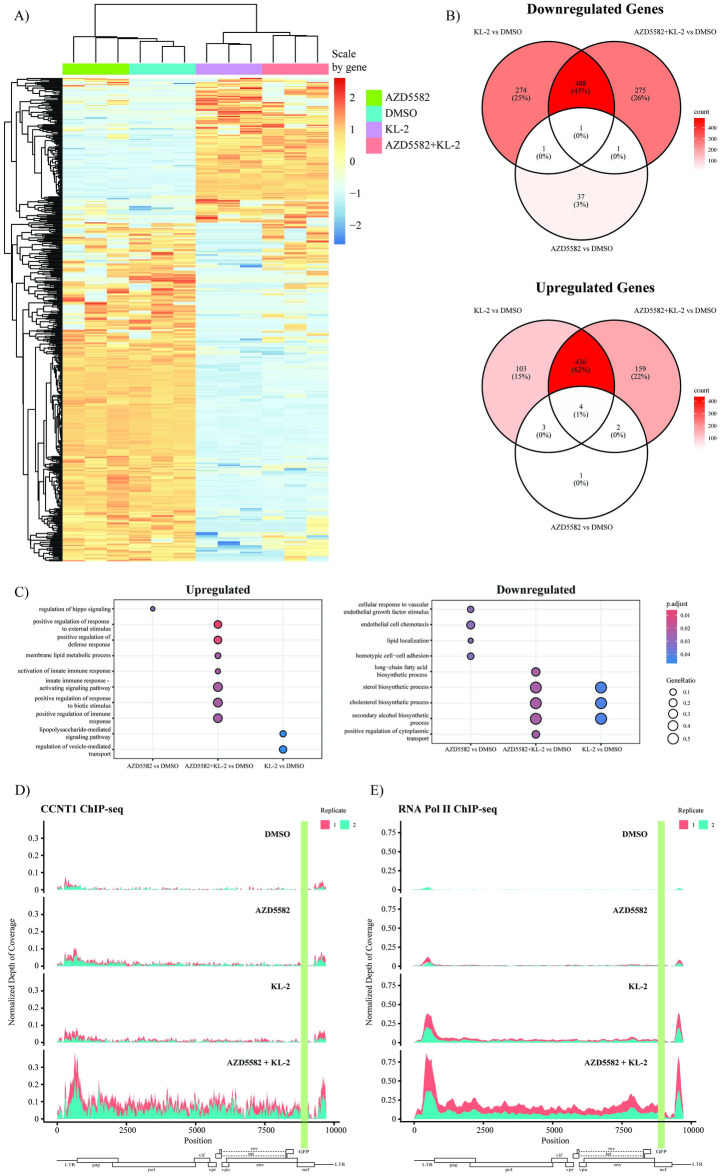
KL-2 increases the occupancy of P-TEFb and RNA Pol II in the proviral gene body. **A)** Heatmap of transcript levels after variance stabilization transformation for each differentially expressed gene following treatment of J-Lat 5A8 cells with DMSO, KL-2, AZD5582, or a combination of AZD5582 and KL-2 for 48 hours (3 biological replicates per treatment). Values are scaled by gene. **B)** Venn diagrams illustrating the number of significantly downregulated (top) and upregulated (bottom) genes that are unique or shared between treatments as compared to the DMSO control. **C)** Gene Ontology (GO) analysis of significantly upregulated (left) and downregulated (right) genes in each treatment condition relative to the DMSO control. The size of the circle indicates the proportion of differentially expressed genes found in each functional category and the color of the circle indicates adjusted p-value. **D)** Sequencing read depth normalized to an equally bounded internal control over the integrated HIV-1 provirus in J-Lat 5A8 cells following CCNT1 or **E)** RNA Pol II ChIP-Seq. Viral open reading frames are indicated below with the location of the integrated GFP reporter indicated in green. Reads from two independent biological replicates are overlaid.

Inhibition of P-TEFb binding to SECs by KL-2 enhanced the HIV-1 latency reactivation potential of several distinct classes of LRAs, but is likely to also cause broad transcriptional changes at SEC-regulated genes. To better understand the effect of KL-2 on the transcriptome, we performed bulk mRNA sequencing (RNA-Seq) on J-Lat 5a8 cells treated with DMSO, AZD5582, KL-2, or AZD5582 plus KL-2 for 48 hours. The transcriptome of cells treated with AZD5582 alone closely resembled that of the DMSO-treated cells (Fig 4A) with only 50 differentially expressed genes (DEGs) (40 downregulated and 10 upregulated; Fig 4B). Treatment with KL-2 resulted in more broad transcriptional changes with more downregulated than upregulated DEGs (764 versus 546, respectively). The cells treated with AZD5582 plus KL-2 exhibited the most transcriptional changes (765 downregulated and 6012 upregulated DEGs), a majority of which were shared with the KL-2 alone. However, the combination of AZD5582 and KL-2 also resulted in the unique upregulation (n = 275) and downregulation (n = 159) of a subset of genes, similar to what was observed with the integrated provirus. Functional enrichment analysis of gene ontology (GO) terms revealed that KL-2 suppressed genes were largely involved in biosynthetic processes corresponding to Myc-regulated pathways as previously reported (Fig 4C) [32,51]. KL-2 in combination with AZD5582 suppressed a similar subset of genes, but also increased expression of several genes involved in the innate immune response to infection, though it remains unclear if this is a direct result of the drug treatment or an indirect effect of proviral reactivation.

The downregulation of several Myc-regulated pathways is consistent with the reported function of KL-2 in disrupting SEC-mediated gene transcription. We hypothesize that the release of P-TEFb from these complexes would allow for its recruitment to sites of proviral transcription where it would facilitate RNA Pol II pause release into the gene body. To test this directly, we performed CCNT1 and RNA Pol II Chromatin Immunoprecipitation Sequencing (ChIP-seq) in J-Lat 5a8 cells treated with either DMSO, KL-2, AZD5582, or KL-2 plus AZD5582 and analyzed occupancy along the provirus relative to an equally bounded internal control (Fig 4D and 4E). The CCNT1 CHIP-Seq data revealed very little P-TEFb along the proviral genome in the DMSO control cells with only slight increases in P-TEFb occupancy at the promotor and in the gene body upon KL-2 or AZD5582 treatment (Fig 4D). Similarly, the RNA Pol II CHIP-Seq data revealed very little RNA Pol II along the proviral genome in the DMSO control and AZD5582 treated cells (Fig 4E). In contrast, treatment with KL-2 alone resulted in a more substantial increase in RNA Pol II at the promoter as well as a slight increase throughout the proviral gene body. Only the combination of KL-2 and AZD5582, however, resulted in a striking increase in both P-TEFb and RNA Pol II throughout the proviral gene body (Fig 4D and 4E).

Taken together, these data are consistent with a model in which SEC disruption promotes P-TEFb recruitment to sites of nascent proviral transcription to promote RNA Pol II pause release. However, the impact of KL-2 on transcriptional initiation and RNA Pol II recruitment to the proviral promoter are suggestive of a potential secondary mechanism that may be related to its broader impacts on the cellular transcriptome.

### Assessing the Tat dependency of KL-2 latency reversal

**Fig 5:**
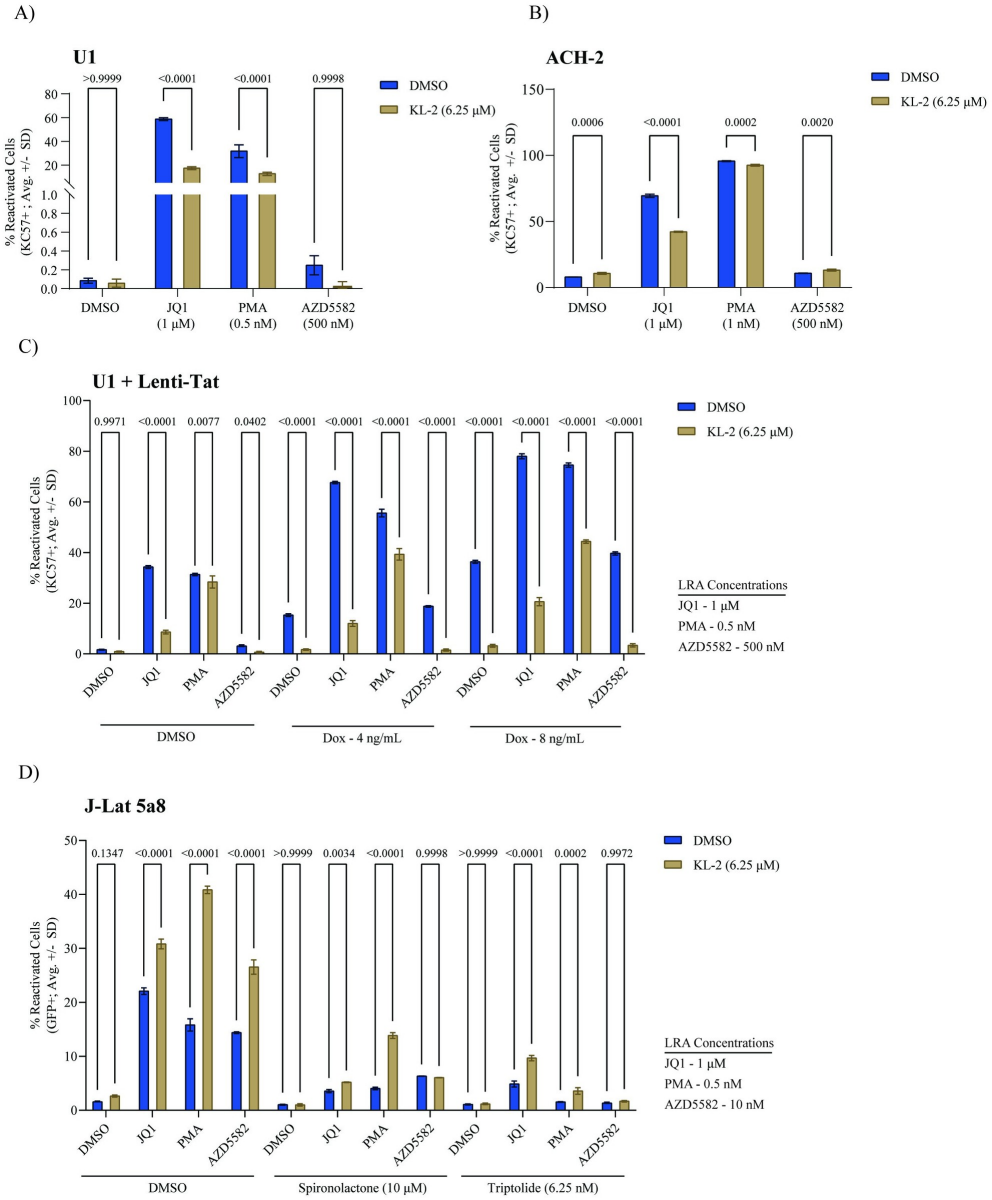
KL-2 latency reversing activity is Tat- and cell line-dependent. **A)** Percent reactivated (KC57-FITC+) U1 cells after 48 hours of treatment with indicated compounds in the presence or absence of KL-2. **B)** Percent reactivated (KC57-FITC+) ACH-2 cells after 48 hours of treatment with indicated compounds in the presence or absence of KL-2. **C)** Percent reactivated (GFP+) J-Lat 5a8 cells after 48 hours of treatment with indicated compounds in the presence or absence of KL-2 and the Tat inhibitors Spironolactone and Triptolide. **D)** Percent reactivated (KC57-FITC+) Lenti-Tat U1 cells after 48 hours of treatment with indicated compounds in the presence or absence of KL-2 and with Tat induction at three different concentrations of doxycycline (0, 4, and 8 ng/mL). For all panels, the data represent the average ± standard deviation of technical triplicates; statistics were calculated by two-way ANOVA with Sidak’s Multiple Comparison test.

The SEC is dispensable for HIV-1 replication in primary CD4+ T cells, and release of P-TEFb from the SEC promotes latency reversal in J-Lat cells, suggesting that P-TEFb is recruited to paused RNA Pol II at proviral integration sites in an SEC-independent manner in these models. This is most likely through the viral accessory protein Tat, which can recruit P-TEFb directly to sites of nascent viral transcription through an interaction with the TAR stem-loop on the 5’ end of viral transcripts (though alternate mechanisms for P-TEFb recruitment have been described). To assess the Tat-dependency of latency reactivation, we first turned to two cell line models of latency that lack a functional Tat-TAR axis: U1 cells and ACH-2 cells. The U1 cell line is a U937-based monocytic cell line that harbors two copies of the HIV-1 provirus, one of which expresses no Tat due to the lack of a start codon and the second of which encodes a Tat mutant with suboptimal P-TEFb affinity [52]. The ACH-2 cell line is a T cell line with one integrated provirus that encodes a fully functional Tat, but that lacks a functional TAR stem loop [53]. Both cell lines were treated with the same LRA panels as before in the presence and absence of KL-2 for 48 hours. These cell lines lack a fluorescent reporter; therefore, reactivation was monitored by intracellular p24 immunostaining and flow cytometry (viability data in S4A and S4B Fig). JQ1 and PMA treatment resulted in strong reactivation in both cell line models while AZD5582 had minimal effects (Fig 5A and 5B). Treatment with KL-2 alone likewise induced negligible reactivation. In contrast to what was observed in J-Lat lines, however, combinatorial treatment with KL-2 significantly decreased the reactivation potential of JQ1 and PMA in both cell lines (Fig 5A and 5B).

These data suggest that while the SEC may be dispensable for Tat-dependent transcription and reactivation, it may play an essential role in P-TEFb recruitment without functional Tat. If so, the delivery of exogenous Tat to U1 cells might be sufficient to circumvent the need for the SEC, reversing the KL-2 treatment phenotype. To test this hypothesis, we transduced U1 cells with a doxycycline (Dox)-inducible lentiviral construct encoding full-length HIV-1 NL4.3 Tat (referred to as Lenti-Tat), selecting for a pure polyclonal population of transduced cells in puromycin. The U1 + Lenti-Tat cells were treated with a range of Dox concentrations, resulting in a dose-dependent increase in proviral reactivation (S4C Fig). U1 + Lenti-Tat cells were then treated with the same panel of LRAs in the presence or absence of KL-2 in the presence of either 0, 4, or 8 ng/mL Dox (Fig 5C; viability data in S4D Fig). Without Tat induction, KL-2 suppressed JQ1- and PMA-mediated reactivation as before (Fig 5A). Tat induction by Dox was sufficient to induce a basal level of reactivation, which was almost entirely reversed by KL-2 treatment (Fig 5C). JQ1 and PMA still induced reactivation above the Tat-induced baseline, but retained sensitivity to KL-2, while AZD5582 failed to reactivate above baseline even in the presence of Tat. These data suggest that the presence of Tat alone is not sufficient to dictate the impact of KL-2 on latency reversal, which is likely additionally dependent on other factors including P-TEFb availability and distribution. Indeed, immunoblotting for P-TEFb (CDK9 and CCNT1) and SEC (AFF4) components revealed that U1 cells have lower steady-state levels of P-TEFb and AFF4 compared to J-Lat cells at baseline, which might alter the efficacy and secondary effects of an SEC disrupter (S4E Fig).

Given these considerations, we next asked if Tat was necessary for the enhanced reactivation phenotype of KL-2 in the J-Lat 5A8 cell line model using two previously reported Tat-dependent transcription inhibitors: Spironolactone and Triptolide (Fig 5D; viability data in S4F Fig). Spironolactone induces the degradation of the XBP helicase, a component of the TFIIH initiation complex, which inhibits Tat-dependent transcription [54], while Triptolide promotes proteasomal degradation of the Tat protein itself [55]. In the presence of the DMSO control, KL-2 alone had little effect, but enhanced the latency reactivation activity of JQ1, PMA, and AZD5582 as observed previously (Fig 3D). Both Tat inhibitors dramatically reduced the reactivation potency of each LRA, in some cases to near baseline levels. Treatment with KL-2, however, still enhanced reactivation in combination with JQ1 and PMA, though not in combination with AZD5582, suggesting that this LRA may be uniquely dependent on Tat and/or the SEC for P-TEFb recruitment (Fig 5D).

Spironolactone and Triptolide do not directly target Tat and are known to have several additional impacts on the cellular transcriptional machinery, including degradation of RNA Polymerase II and inhibition of NF-kB signaling [56–59]. While didehydro-cortistatin A has been reported to be a more specific inhibitor of Tat, we were unable to source or synthesize the compound for testing [60]. Therefore, as an alternate approach, we next tested the impact of KL-2 on latency reversal in a pair of J-Lat cell lines that have an integrated HIV-1 LTR-driven GFP reporter that either do (A2 cells) or do not (A72 cells) express Tat [61,62]. Each cell line was treated with the same panel of LRAs as before in the presence and absence of KL-2 for 48 hours. In the Tat-expressing J-Lat A2 cells, KL-2 alone had no effect while each LRA resulted in robust reactivation (S4G Fig). As expected, KL-2 enhanced JQ1 and PMA activity, but surprisingly decreased AZD5582 activity slightly. In contrast, J-Lat A72 cells had a high level of basal GFP expression, responded only mildly to any of the LRAs, and exhibited a slight increase in reactivation in the presence of KL-2 in all conditions (S4H Fig). Taken together, these data suggest that while SEC disruption may enhance viral transcription and latency reactivation in most models, it may also promote latency in certain cellular contexts that is not fully explained by the presence or absence of Tat expression.

### KL-2 increases HIV-1 viral transcripts in PBMCs from aviremic patients

**Fig 6:**
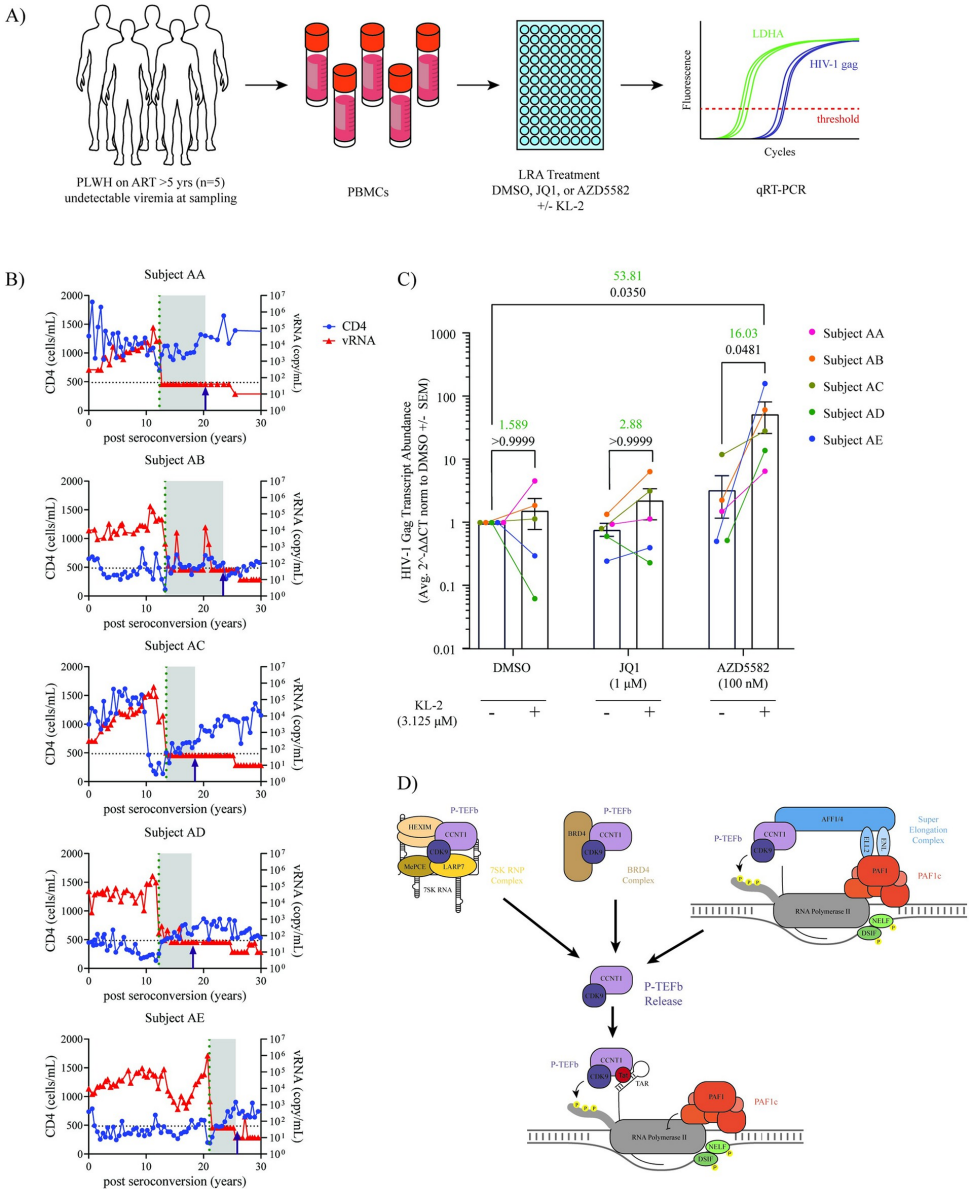
KL-2 enhances latency reversing agent activity in PBMCs from virally suppressed people living with HIV. **A)** Experimental schematic of the latency reversal assay using PBMCs from five HIV-1 patients with undetectable viral loads. Intracellular *gag* transcript levels were measured by qRT-PCR after treatment with JQ1, PMA, or AZD5582 in the presence or absence of KL-2. **B)** Overlaid line graphs depicting the CD4 count (cells/mL, blue) and levels of viral RNA (copies/mL, red) in each subject over time. The dotted line shows the time of antiretroviral therapy initiation, and the time of the blood draw used for this assay is depicted by an arrow. **C)** Relative transcript levels of HIV-1 *gag* relative to human *LDHA* in patient PBMCs (n = 5 donors) after 48 hours of treatment with the indicated compounds in the presence or absence of KL-2. Data represent the average 2^-ΔΔCt [(Ct_gag_-Ct_LDHA_)LRAs— (Ct_gag_-Ct_LDHA_)_DMSO_] ± SEM of technical triplicates. Statistics were calculated using a two-way ANOVA with Sidak’s Multiple Comparison Test. The fold change in mean 2^-ΔΔCt is shown above for relevant comparisons in green text with corresponding p-values shown below in black text. **D)** Model of cellular P-TEFb reservoirs whose disruption have demonstrated activity in HIV-1 latency reversal.

Blocks to transcriptional elongation have been shown to contribute to HIV-1 latency maintenance in cells from virally suppressed people living with HIV (PLWH) [13,14]. The release of P-TEFb sequestration from BRD4 using bromodomain inhibitors (such as JQ1) has proven effective at reactivating viral gene expression in peripheral blood mononuclear cells (PBMCs) from these patients [63]. We hypothesized that KL-2 would likewise reactivate viral gene expression in patient PBMCs. To test this, cryopreserved PBMCs were obtained from five PLWH enrolled in the Northwestern University Clinical Research Site for the MACS/WIHS Combined Cohort Study (MWCCS) (Fig 6A). The selected PLWH had been virally suppressed with ART for more than five years with undetectable HIV-1 plasma levels at the time of blood draw (<50 copies/ml) (Fig 6B). Cells from these patients were treated for 48 hours with DMSO, JQ1, or AZD5582 alone or in combination with KL-2. RNA was isolated from the treated cells and reactivation of viral gene expression was measured by qRT-PCR for HIV-1 *gag* (relative to the human housekeeping gene *LDHA*, Fig 6C).

Similar to what was observed in our cell line models, treatment with KL-2 alone resulted in minimal latency reactivation with only a slight increase in *gag* expression in three out of the five donors, with the mean transcript levels increasing 1.5-fold (not statistically significant, Fig 6C). Treatment with JQ1 alone was not sufficient to induce *gag* expression in these donors, though the addition of JQ1 and KL-2 together resulted in a 2.88-fold increase over JQ1 treatment alone (not significant, Fig 6C). AZD5582 treatment alone resulted in a roughly 3.36-fold increase in *gag* expression over the DMSO control. However, KL-2 significantly enhanced the ability of AZD5582 to reactivate HIV-1 transcription across all donors with a marked 53-fold increase over the DMSO only control and a 16-fold increase compared to AZD5582 alone (Fig 6C). Taken together, these data demonstrate that the release of P-TEFb from cellular SECs using the small molecule KL-2 can enhance the effects of a range of LRAs in cell line models of HIV-1 latency as well as improve transcriptional reactivation by AZD5582 in primary PBMCs from HIV-1 patients.

## Discussion

In this study, we demonstrate a potential new strategy for enhancing HIV-1 latency reversal through the release of P-TEFb from the cellular pool of SECs. We show that KL-2, a small molecule inhibitor of the interaction between the SEC and P-TEFb, is sufficient to enhance viral transcription in primary CD4+ T cells and can synergistically enhance the activity of other LRAs in certain cell line models of latency. Finally, we demonstrate that KL-2 can increase HIV-1 *gag* expression in PBMCs from PLWH on suppressive ART, most notably in combination with the non-canonical NF-kB agonist, AZD5582. We propose a model in which KL-2 release of P-TEFb from the cellular pool of SECs enhances transcriptional elongation of integrated proviruses, akin to BET bromodomain inhibitors and 7SK RNP inhibitors (Fig 6D). These results have several implications for our understanding of viral transcription and future directions.

Latency reversal through the release of P-TEFb from cellular SECs had not been previously explored, likely due to the perceived dependency of viral transcription on the SEC. This effect has been supported by biochemical purifications of HIV-1 Tat from human cell lines that revealed interactions with a larger SEC [41,42], as well as by genetic knock-down experiments in cell lines showing a decrease in Tat-dependent transcription upon SEC component depletion [64,65]. However, given that both the SEC and Tat recruit P-TEFb to sites of nascent transcription, they share some functional redundancy. We found that knock-out of SEC components from activated, primary CD4+ T cells from 12 independent donors did not inhibit viral replication, suggesting that the SEC is not required in this cellular context. This result was independently verified using KL-2, which inhibits the interaction between CCNT1 (P-TEFb) and AFF1/4 (of the larger SEC). Notably, disruption of the SEC using KL-2 resulted in significant increases in HIV-1 replication in primary CD4+ T cells whereas genetic knockout of most SEC members had minimal to no impact on replication. One potential explanation for this difference is that genetic knockout results in the ablation of SEC assembly and relocalization of P-TEFb into other complexes at steady-state whereas chemical perturbation by KL-2 results in the release of P-TEFb from SECs that are continually being formed. Understanding the dynamics of P-TEFb distribution and relocalization upon different types of perturbation is an ongoing area of investigation.

While we expected KL-2 to enhance the transcriptional elongation of integrated proviruses due to the release of P-TEFb from cellular SECs, we also saw increases in transcriptional initiation as measured by qRT-PCR for TAR transcript levels. Likewise, in J-Lat 5A8 cells, we saw increases in transcriptional elongation, transcriptional initiation, and in RNA Pol II recruitment to the proviral promoter when KL-2 was added, even though KL-2 addition alone was not sufficient for reactivation as measured by GFP positivity in this model. This finding suggests that either KL-2 has secondary effects not mediated by P-TEFb or that the redistribution of P-TEFb away from SECs has secondary effects that could impact transcriptional initiation at proviral integration sites. BET bromodomain inhibitors have also been reported to increase HIV-1 transcriptional initiation [12,13], though these effects have been suggested to occur through modulation of the epigenetic regulatory functions of these proteins [66]. Other reports have indicated that P-TEFb mediated release of paused RNA Pol II can result in enhanced transcriptional initiation and even RNA Pol II recruitment simply by increasing the number of transcribing polymerases [67]. Still, this connection between transcriptional elongation and initiation has yet to be fully understood in the context of HIV-1 transcription.

While KL-2 was sufficient to boost viral replication in activated, primary CD4+ T cells, in and of itself it displayed minimal reactivation potential in both cell line models of latency and in PBMCs from PLWH on suppressive ART. This finding is similar to our recent report of a novel inhibitor of the PAF1 complex (iPAF1C) that had minimal activity on its own, but greatly enhanced the reactivation potential of other LRAs [16]. Both cases highlight the multifaceted nature of the blocks to viral gene expression that underlie the latent state as well as the limitations to single agent drug screening to identify promising, next-generation LRAs. Combinatorial approaches to dissect the genetic underpinnings of HIV-1 latency and discover new, synergistic drug interactions should be prioritized.

While KL-2 alone failed to significantly increase HIV-1 *gag* transcript levels in patient PBMCs, in combination with AZD5582 it resulted in a 16-fold increase over AZD5582 treatment alone and a 53-fold increase over the DMSO control. Crosswise dose titrations in J-Lat 5A8 cells showed a strong synergistic potential between AZD5582 and KL-2. This is consistent with reports of robust synergy between AZD5582 and P-TEFb release through BET bromodomain inhibition [12]. Notably, the BET bromodomain inhibitor JQ1 showed minimal reactivation activity in our patient PBMCs, even in the presence of KL-2, in contrast to our cell line data. This finding could reflect stochastic differences driven by variations in patient characteristics, integration site, chromatin state, transcription factor availability, etc. [13]. Future work will compare P-TEFb release from SECs to release from other cellular reservoirs, such as BRD4 or the 7SK RNP. Recent studies have shown that post-translational modifications of P-TEFb, most notably phosphorylation of CDK9 Serine 175 and Threonine 186, can drive inclusion into different complexes and may strongly influence bioavailability and activity [40, 68–70]. Therefore, it is possible that disruption of complexes housing ‘active’ P-TEFb is a more direct route to redirecting P-TEFb activity.

This is not to say that the SEC is never required for viral transcription. In latency model cell lines that lacked a functional Tat (U1 cells) or TAR stem loop (ACH-2 cells), KL-2 inhibited the reactivation potential of several LRAs, suggesting that viral transcription may be more dependent on the SEC when Tat is either defective or not expressed. We attempted to test this by inhibiting Tat in the J-Lat 5A8 model cell line using two previously described Tat-dependent transcription inhibitors, Triptolide and Spironolactone. While both compounds reduced LRA efficacy, KL-2 still boosted the activity of JQ1 and PMA, but not AZD5582. This suggests that P-TEFb can be recruited to proviral integration sites in a Tat and SEC-independent manner upon PMA or JQ1 treatment, potentially through a transcription factor such as NF-kB. The inability of AZD5582 to do so suggests that non-canonical NF-kB activation does not recruit the same milieu of transcription factors, making it uniquely Tat or SEC dependent. This is consistent with the complete lack of activity of AZD5582 in cell line models lacking functional Tat/TAR activity and may underlie the remarkable synergy between non-canonical NF-kB agonists and agents that release P-TEFb [12]. Additionally, triptolide has been characterized outside of HIV-1 transcription in its ability to prevent RNA Pol II reinitiation following pausing through inhibition of xeoderma pigmentosum group B-complementing protein (XPB) and is often used as a tool compound for measuring the fate of paused RNA Pol II at different time points [51,71]. With this in mind, it is possible that compounds JQ1 and PMA result in *de novo* recruitment of RNA Pol II thereby increasing transcriptional initiation whereas AZD5582 may be more reliant on RNA Pol II pause-release.

To further explore the Tat dependency of KL-2, we tried to rescue Tat function in the U1 cell line using a Dox-inducible system. We hypothesized that by providing Tat, the SEC would no longer be required for viral gene expression such that SEC disruption by KL-2 would enhance reactivation as seen in the J-Lat models. While Tat induction itself was sufficient for reactivation, this reactivation was completely abolished by the addition of KL-2. Even when Tat was minimally induced and other LRAs were added, KL-2 still inhibited reactivation, suggesting that additional factors—such as steady-state levels of P-TEFb, integration site, epigenetic factors, or transcription factor availability—may drive SEC dependency besides just the presence or absence of Tat. Indeed, SEC disruption by KL-2 in the original report of the inhibitor demonstrated an outsized impact on Myc-dependent transcription [32], suggesting that additional factors driving the SEC dependency of proviral transcription have yet to be described. Regardless, the dual-acting nature of KL-2 in enhancing latency reactivation in some circumstances (*i*.*e*., if Tat is present) and inhibiting latency reactivation in others (*i*.*e*., when the cell state dictates SEC dependency) presents a unique opportunity to leverage the heterogenous nature of the latent reservoir to both reverse and promote latency.

Taken together, our results indicate that release of P-TEFb from cellular SECs is a novel mechanism for promoting HIV-1 viral transcription during both active and latent infection. We demonstrated the enhancement of latency reversal in multiple latent cell line models and in primary PBMCs from PLWH on suppressive ART. This work demonstrates the importance of increasing the production or availability of free P-TEFb for recruitment to viral loci as a powerful strategy for bolstering current LRAs, most notably non-canonical NF-kB agonists. Due to the heterogeneity of blocks to viral replication in the latent reservoir, it is likely that combinatorial LRA treatments will be the best strategy for potent latency reversal moving forward. Further efforts are needed to understand the intracellular distribution of active P-TEFb to characterize the most critical reservoir to target to enhance transcription. Additionally, our work demonstrates that disruption of SECs could enhance latency reversal or promote the maintenance of latency depending on the cellular context. Understanding the mechanism that controls this molecular switch would aid in understanding whether SEC disruptors could be a viable dual-acting molecule to aid in finding a functional cure for HIV-1 infection.

## Methods

### Ethics statement

Primary human CD4+ T cells from anonymous, healthy donors were isolated from leukoreduction chambers provided by a commercial vendor (STEMCELL Technologies). These were provided without any identifying information and did not require we attain written informed consent. PBMCs from people living with HIV were provided from study subjects enrolled in the Northwestern University Clinical Research Site for the MACS/WIHS combined cohort study (MWCCS). All of these participants provided written informed consent. The Institutional Review Board of Northwestern University approved the study (STU00022906-CR0008) with most recent approval date of May 16, 2022.

### CD4+ T cell isolation

Primary human CD4+ T cells from healthy donors were isolated from leukoreduction chambers after Trima apheresis (STEMCELL Technologies). PBMCs were isolated by Ficoll centrifugation. Bulk CD4+ T cells were subsequently isolated from PBMCs by magnetic negative selection using an EasySep Human CD4+ T cell isolation kit (STEMCELL Technologies; per the manufacturer’s instructions). Isolated CD4+ T cells were suspended in RPMI 1640 (Sigma-Aldrich) supplemented with 5 mM HEPES (Corning), 1% penicillin-streptomycin (50 mg/ml; Corning), 5 mM sodium pyruvate (Corning), and 10% HI FBS (Gibco). Media were supplemented with interleukin-2 (IL-2; 20 IU/ml; Miltenyi) immediately before use. For activation, bulk CD4+ T cells were immediately plated on anti-CD3–coated plates coated for 2 hours at 37 °C with anti-CD3 (20 mg/ml) (UCHT1; Tonbo Biosciences) in the presence of soluble anti-CD28 (5 mg/ml; CD28.2; Tonbo Biosciences). Cells were stimulated for 72 hours at 37 °C and 5% CO_2_ before treatment with KL-2.

### crRNP production

Lyophilized crRNA and tracrRNA (Dharmacon) was resuspended at a concentration of 160 μM in 10 mM Tris-HCL (7.4 pH) with 150 mM KCl. Cas9 ribonucleoproteins (RNPs) were made by incubating 5 μL of 160 μM crRNA (Horizon) with 5 μL of 160 μM tracrRNA for 30 minutes at 37 °C, followed by incubation of the gRNA:tracrRNA complex product with 10 μL of 40 μM Cas9 (UC Berkeley Macrolab) to form RNPs. Five 3.5 μL aliquots were frozen in Lo-Bind 96-well V-bottom plates (E&K Scientific) at −80 °C until use. For synthesis of multiplexed RNPs, four independent crRNA targeting the same gene were mixed at a 1:1:1:1 ratio prior to addition of the tracrRNA as above.

### CRISPR RNA sequences

crRNA was ordered from Horizon Discovery using either the catalog numbers for predesigned guide sequences or custom guide sequences as indicated in Table 1 below.

**Table 1:** CRISPR guide RNA sequences or catalog numbers.

*Gene*	*Guide #1*	*Guide #2*	*Guide #3*	*Guide #4*	*Guide #5*
*CCNT1*	CM-003220-01				
*CDK9*	AGATCGGCCAAGGCACCTTC				
*AFF1*	CM-020074-01	CM-020074-02	CM-020074-03	CM-020074-04	CM-020074-05
*AFF4*	CM-020276-01	CM-020276-02	CM-020276-03	CM-020276-04	CM-020276-05
*ELL2*	CM-008177-01	CM-008177-02	CM-008177-03	CM-008177-04	CM-008177-05
*ENL*	TAGGTGATGGTAATGCCCGA	GGTGGAGGAGTCCAACTCAG	GCTGGATGTCACATTGCTCG	AAGAAGACCAAACCATCCCA	
*AF9*	AGCTTTCCTAGGCCAAAAAG	CAGCGGAGGTGATTCACTGG	GGATCCCAATGATTCAGATG	GTACGAACACCATCCAGTCG	
*CXCR4*	GAAGCGTGATGACAAAGAGG				
*CYPA*	AGGTCCCAAAGACAGCAGGT				
*Non-targeting*	U-007503-20				

### CD4+ T cell CRISPR knockouts

Following CD4+ T cell isolation and stimulation, as above, cells were counted, centrifuged at 400 × g for 5 minutes, the supernatant was removed by aspiration, and the pellet was resuspended in 20 μL of supplemented room-temperature P3 electroporation buffer (Lonza) per reaction. Each reaction consisted of 1 × 10^6^ cells, 3.5 μL of RNP, and 20 μL of electroporation buffer. The cell suspension was then gently mixed with thawed RNP and aliquoted into a 96-well electroporation cuvette for electroporation with the 4D 96-well shuttle unit (Lonza) using pulse code EH-115. Immediately after electroporation, 80 μL of prewarmed media without IL-2 was added to each well and cells were allowed to rest for at least 30 minutes in a 37 °C cell culture incubator. Subsequently, cells were moved to 96-well flat-bottom culture plates prefilled with 100 μL of warm complete media with IL-2 at 40 IU/mL (for a final concentration of 20 IU/mL) and anti-CD3/anti-CD2/anti-CD28 beads (T cell Activation and Stimulation Kit, Miltenyi) at a 1:1 bead:cell ratio per the manufacturer’s instructions.

### Whole cell protein lysate preparation

Whole cell lysates were prepared by suspension of cell pellets (Typically ~150,000 cells) directly in 2.5x Laemmli Sample Buffer followed by denaturization at 98 °C for 30 minutes.

### Affinity purification of endogenous CCNT1

10 million cells were pelleted, washed with PBS, and subsequently resuspended in 1 mL of lysis buffer (0.5% NP40, 50 mM Tris–HCl pH 7.4; 150 mM NaCl, 1 mM EDTA, cOmplete protease (Roche) and PhosSTOP phosphatase (Roche) inhibitors). Samples were then rotated at 4 °C for 30 minutes and transferred to -80 °C for at least 30 minutes to complete cell lysis. Following lysis, samples were thawed on ice and centrifuged in a prechilled microcentrifuge at 3500 x g for 20 minutes to remove cellular debris. Lysates were then precleared by incubation for 1 hour at 4 °C while rotating with 50 μL protein A agarose beads (Cell Signaling Technologies, Cat #9863). CCNT1 antibody (Cell Signaling Technologies, Cat #81464) was then added to precleared lysates at 1:100 and incubated with rotation overnight at 4 °C. After incubation, 50 μL of fresh Protein A agarose beads were added to each sample and incubated with rotation for 1–3 hours at 4 °C. Samples were then washed twice with IP buffer (50 mM Tris–HCl pH 7.4; 150 mM NaCl, 1 mM EDTA) and supernatant was removed. 100 μL 2.5X Laemmli Sample Buffer was then added to the washed beads and denatured at 98 °C for 30 minutes.

### Immunoblotting

Samples were run on 4–20% Tris-HCl SDS-PAGE gels (BioRad Criterion) at 90 V for 40 minutes followed by separation at 150 V for 85 minutes. Proteins were transferred to PVDF membranes by electrotransfer (BioRad Criterion Blotter) at 90 V for 2 hours. Membranes were blocked in 5% milk in DPBS, 0.1% Tween-20 or 5% BSA in DPBS, 0.1% Tween-20 for 1 hour prior to primary antibody incubation overnight at 4 °C. Anti-rabbit or anti-mouse IgG horseradish peroxidase (HRP)-conjugated secondary antibodies (1:20000, polyclonal, Jackson ImmunoResearch Laboratories, Cat. Nos. 111-035-003 and 115-035-003) were detected using Pierce ECL Western Blotting Substrate (ThermoFisher) on iBright (Thermofisher) blot scanner. Blots were incubated in a 1xPBS, 0.2 M glycine, 1.0% SDS, 1.0% Tween-20, and pH 2.2 stripping buffer before reprobing.

### Primary antibodies

Details for each primary antibody used in this study are provided in Table 2 below, including the target protein, manufacturer, animal, catalog number, and URL.

**Table 2:** Primary antibody details.

Protein	Manufacturer	Animal	Catalog #	URL
CCNT1	CST	Rabbit	81464	https://www.cellsignal.com/products/primary-antibodies/cyclin-t1-d1b6g-rabbit-mab/81464
CDK9	CST	Rabbit	2316	https://www.cellsignal.com/products/primary-antibodies/cdk9-c12f7-rabbit-mab/2316
AFF1	Bethyl	Rabbit	A302-344A	https://www.fortislife.com/products/primary-antibodies/rabbit-anti-af4-antibody/BETHYL-A302-344
AFF4	Proteintech	Rabbit	14662-1-AP	https://www.ptglab.com/products/AFF4-Antibody-14662-1-AP.htm
ELL2	Bethyl	Rabbit	A302-505A	https://www.fortislife.com/products/primary-antibodies/rabbit-anti-ell2-antibody/BETHYL-A302-505
AF9	Bethyl	Rabbit	A300-595A	https://www.fortislife.com/products/primary-antibodies/rabbit-anti-af9-antibody/BETHYL-A300-595
ENL	CST	Rabbit	14893	https://www.cellsignal.com/products/primary-antibodies/mllt1-enl-d9m4b-rabbit-mab/14893
Rabbit IgG	CST	Rabbit	3900	https://www.cellsignal.com/products/primary-antibodies/rabbit-da1e-mab-igg-xp-174-isotype-control/3900
β-Actin	CST	Mouse	3700	https://www.cellsignal.com/products/primary-antibodies/b-actin-8h10d10-mouse-mab/3700

### Preparation of virus stocks for infection of primary CD4+ T cell cultures

Replication-competent reporter virus stocks were generated from an HIV-1 NL4.3 molecular clone in which GFP had been cloned behind an IRES cassette following the viral *nef* gene (NIH AIDS Reagent Program, catalog no. 11349). Briefly, 10 μg of the molecular clone was transfected (PolyJet; SignaGen) into 5 × 10^6^ human embryonic kidney (HEK) 293T cells (ATCC, CRL-3216) according to the manufacturer’s protocol. 25 mL of the supernatant was collected at 48 and 72 hours and then combined. The virus-containing supernatant was filtered through 0.45-mm polyvinylidene difluoride filters (Millipore) and precipitated in 8.5% polyethylene glycol [average molecular weight (Mn), 6000; Sigma-Aldrich] and 0.3 M NaCl for 4 hours at 4 °C. Supernatants were centrifuged at 3500 rpm for 20 minutes, and the concentrated virus was resuspended in 0.25 ml of PBS for a 100X effective concentration. Aliquots were stored at −80 °C until use.

### HIV-1 Infection of CRISPR-edited primary CD4+ T cell cultures

Edited primary CD4+ T cells were plated into a 96-well, round-bottom plate at a cell density of 1 × 10^5^ cells per well and cultured overnight in 200 μL of complete RPMI 1640 as described above in the presence of IL-2 (20 IU/ml) and 2.5 μL of concentrated virus stock. Cells were cultured in a dark humidified incubator at 37 °C and 5% CO_2_. On days 2 and 5 after infection, 75 μL of each culture was removed and mixed 1:1 with freshly made 2% formaldehyde in PBS (Sigma-Aldrich) and stored at 4 °C for analysis by flow cytometry. Cultures were supplemented with 75 ml of complete IL-2–containing RPMI 1640 medium and returned to the incubator.

### HIV-1 infection of primary CD4+ T cell cultures treated with KL-2

Activated primary CD4+ T cells were plated into a 96-well, round-bottom plate at a cell density of 1 × 10^5^ cells per well and cultured overnight in 200 μL of complete RPMI 1640 as described above in the presence of IL-2 (20 IU/mL) with different concentrations of KL-2 or equivalent volumes of DMSO. The next day, 2.5 μL of concentrated virus stock was added to each well. Cells were cultured in a dark humidified incubator at 37 °C and 5% CO_2_. On days 2 and 5 after infection, 75 μL of each culture was removed and mixed 1:1 with freshly made 2% formaldehyde in PBS (Sigma-Aldrich) and stored at 4 °C for analysis by flow cytometry. Cultures were supplemented with 75 mL of complete IL-2–containing RPMI 1640 medium and returned to the incubator.

### HIV-1 infection of microglial CHME3-4x4 cell cultures treated with KL-2

CHME3-4x4 cells were plated into a 96-well, flat-bottom plate at a cell density of 10,000 cells per well and cultured overnight in 200 uL of DMEM + 10% FBS. After overnight seeding, the media was removed and replaced with DMEM + 10% FBS and different concentrations of KL-2 or equivalent volumes of DMSO. The next day, the cells were infected with 2.5 uL of HIV-1 NL4.3 containing a nano-luciferase reporter in place of Nef (Nef:Nano-Luc). Two days following infection, the cell culture media was removed and Nano-Luc production was measured using the Nano-Glo Luciferase Assay System (Promega, Cat# N1110). In parallel, an identical plate of CHME3-4x4 cells treated with KL-2 was cultured as described above. On the same day as the infection analysis, the identical plate was analyzed for viability using the CellTiter Glo Luminescent Viability Assay System (Promega, Cat# G7572).

### Latent cell line drug treatment assays

J-Lat 5A8, J-Lat 11.1, J-Lat 6.3, J-Lat A72, U1, and ACH-2 cell lines were plated in 96-well flat bottom plates at a density of 50,000 cells/200 μL supplemented RPMI 1640. Cells were DMSO-treated or treated with KL-2 (6.25 μM), JQ1, PMA, and AZD5582 for 48 hours at the concentrations indicated below in Table 3. Cells were then washed in PBS and resuspended in PBS + 1% formaldehyde and fixed for 30 minutes. Analysis was performed by flow cytometry gating on GFP-positive cells for J-Lat cell lines.

**Table 3:** Final concentrations for latency reversing agents used for each cell line.

Cell Line	JQ1	PMA	AZD5582
J-Lat 5a8	1 μM	0.5 nM	10 nM
J-Lat 6.3	1 μM	0.5 nM	10 nM
J-Lat 11.1	1 μM	0.5 nM	10 nM
J-Lat A72	1 μM	0.5 nM	10 nM
U1	1 μM	0.5 nM	500 nM
ACH-2	1 μM	1 nM	500 nM

### Immunostaining

Extracellular staining was performed on uninfected, live primary T cell populations with anti-CD4-PE (Miltenyi Biotec, 130-113-225), anti-CXCR4-APC (Miltenyi Biotec, Cat#130-120-708), and anti-CD25-APC (Miltenyi Biotec, Cat#1130-115-535) antibodies according to the manufacturer’s instructions. Briefly, cells were pelleted, and media was removed. Cells were then washed once with DPBS and resuspended in a 1:50 dilution of the appropriate antibodies in MACS buffer (DPBS + 0.5% bovine serum albumin (BSA) and 2 mM EDTA) and incubated for 15 minutes at 4 °C. Cells were then pelleted, washed with MACS buffer, and suspended in DPBS + 1% formaldehyde for 30 minutes prior to flow cytometry.

Viability staining was performed on various cell populations using the amine-reactive dye Ghost Red 710 (Tonbo, Cat#13-0871-T100) according to manufacturer’s instructions. Briefly, cells were pelleted and media was removed. Cells were then washed once with DPBS and resuspended in a ghost red dye solution consisting of 1 μL of ghost red dye per 1 mL of DPBS and incubated for 30 minutes at 4 °C. Cells were then pelleted, washed with MACS buffer, and suspended in DPBS + 1% formaldehyde for 30 minutes prior to flow cytometry.

Intracellular HIV-1 p24 staining was performed on fixed U1 and ACH-2 cells with KC57-FITC (Beckman Coulter, Cat#6604665). After allowing at least 30 minutes for fixation, cells were pelleted and washed with DPBS. Subsequently, cells were suspended in DPBS + 1% BSA, 0.1% w/v saponin and incubated at room temperature for 20 minutes to block and permeabilize. Cells were then spun down to remove the supernatant and incubated for 30 minutes at room temperature in the dark with KC57-FITC at a concentration of 1:50 in DPBS plus 1% BSA, 0.1% saponin w/v. Cells were then pelleted again, washed with PBS plus 1% BSA, and suspended in 1% formaldehyde DPBS for fixation prior to flow cytometry.

### Flow cytometry and analysis of viability/infection/reactivation data

Flow cytometry analysis was performed on an Attune NxT acoustic focusing cytometer (Thermo Fisher Scientific), recording all events in a 40-μL sample volume after one 150 μL of mixing cycle. Data were exported as FCS3.0 files using Attune NxT Software v3.2.0 and analyzed with a consistent template on FlowJo. Briefly, cells were gated for lymphocytes by light scatter followed by doublet discrimination in both side and forward scatter. Cells with equal fluorescence in the BL-1 (GFP) channel and the VL-2 (AmCyan) channel were identified as autofluorescent and excluded from the analysis. A consistent gate was then used to quantify the fraction of remaining cells that expressed the target of interest.

### RNA Isolation and cDNA synthesis

Total RNA isolation from cells (typically 100,000 cells) treated with our LRA panel in the presence or absence of KL-2 was carried out with an RNeasy kit (Qiagen), with the optional on-column deoxyribonuclease I digestion step. The isolated total RNA was eluted in ribonuclease-free water and RNA concentrations were subsequently quantified using a NanoDrop One (Thermo Fisher).

cDNA was synthesized from extracted RNA (typically 100 ng) using the SuperScript IV first strand synthesis system (Invitrogen, Cat#18091050) according to the manufacturer’s instructions. Briefly, a 13 μL reaction mixture was made with random hexamers (1 μL; 50 ng/μL), 10 mM dNTP (1 μL), template RNA (100 ng) and DEPC-treated water. The RNA-hexamer mix was then incubated at 65 °C for 5 minutes before incubating on ice for another minute. Then a mix of 5x SSIV Buffer (4 μL), 100 mM DTT (1 μL), Ribonuclease Inhibitor (μL), and SuperScript IV Reverse Transcriptase (1 μL, 200 U/μL) was added to each sample. The combined reaction mixture was then incubated at 23C for 10 minutes, followed by 50 °C for 10 minutes, and the reaction was inactivated by incubation at 80 °C for 10 minutes. Following complete reverse transcription, residual RNA was cleared by the addition of 1 μL *E*. *Coli* RNase H to each reaction mix and incubated at 37 °C for 20 minutes. The RT products were then stored at -20 °C.

### Analysis of HIV-specific RNA transcripts

Following cDNA synthesis, viral transcripts were assessed by qRT-PCR as described previously [13]. For HIV-1 TAR, the following primers were used: PF: 5′-GTCTCTCTGGTTAGACCAG-3′; PR: 5′-TGGGTTCCCTAGYTAGCC-3′; and probe: 5′-AGCCTGGGAGCTC-3′. HIV-1 Long LTR levels were assessed using the following primers: PF: 5′-GCCTCAATAAAGCTTGCCTTGA-3′; PR: 5′-GGGCGCCACTGCTAGAGA-3′; and probe: 5′-CCAGAGTCACACAACAGACGGGCACA-3. For expression level normalization, *β-Actin* was used (Thermo Fisher Scientific, catalog no. 4331182). Notably, *β-Actin* is an RNA Pol II controlled gene. To ensure that *β-Actin* levels were not being altered by KL-2 induced SEC disruption, we compared *β-Actin* expression to that of the 18S ribosomal subunit, an RNA Pol I controlled gene (Thermo Fisher Scientific, catalog no. 4331182). No statistically significant changes were noted between the two housekeeping genes upon KL-2 treatment, so *β-Actin* was used as the normalization gene for subsequent qPCR analyses. The reaction was performed using TaqMan Fast Advanced Master Mix (Thermo Fisher Scientific, catalog no. 4444553) according to manufacturer’s instructions. Briefly, 10 μL of reaction was mixed using 5 μL of Taqman Master Mix, 0.5 μL of 20x primer probe mix (18 μM of primers and 5 μM probe), 2.5 μL water, and 2 μL of template cDNA. The PCR cycles were as follows: 50 °C for 2 minutes, 95 °C for 20 seconds, followed by 40 cycles of 95 °C for 1 second and 60 °C for 20 seconds.

### Chromatin immunoprecipitation sequencing (ChIP-seq)

ChIP-seq was performed according to a previously published protocol [72]. Briefly, culture media were aspirated and about 20 million to 50 million of cells were washed twice with ice-cold 1× phosphate-buffered saline (PBS; Thermo Fisher Scientific, catalog no. 14190250) and then cross-linked with 1% paraformaldehyde (Thermo Fisher Scientific, catalog no. 28908) for 10 min while shaking at room temperature. The reaction was quenched using 0.2 M glycine (Fisher Scientific, catalog no. BP381-5) for 5 minutes at room temperature followed by centrifugation at 1350 RPM for 5 minutes. Subsequently, cells were gently washed with ice-cold DPBS and centrifuged at 1350 RPM for 5 minutes. For RNA Pol II and CCNT1 ChIP-seq, the chromatin was sonicated using Covaris E220 for 4 minutes using the following sonication conditions: 10% duty cycle, 140 peak intensity power, and 200 cycles per burst. Pulldown of chromatin was carried out overnight at 4°C using the specific antibodies [Rpb1 NTD (D8L4Y, CST catalog no. 14958) rabbit monoclonal antibody for RNA Pol II and CCNT1 (D1B6G, CST catalog no. 81464) rabbit monoclonal antibody]. The next day, Dynabeads protein G (Invitrogen, catalog no. 10004D) were added to the immunoprecipitation mix and incubated at 4 °C for 4 hours. Nonspecific proteins were washed away, and bead-bound proteins were digested using proteinase K (400 mg/ml; Roche, catalog no. 3115828001). Reverse cross-linking was performed at 65 °C overnight while shaking at 1200 RPM. Immunoprecipitated DNA was extracted using phenol-chloroform (Thermo Fisher Scientific, catalog no. 17909) followed by ethanol precipitation and washing. DNA was dissolved in 10 mM tris-HCl (pH 8) and quantified using Qubit. DNA libraries were prepared by the HTP Library Preparation Kit for Illumina (KAPA) and sequenced on NovaSeq 6000 or NovaSeq X (Illumina) in the single-end (SE) mode.

### ChIP-Seq viral assembly

In order to analyze RNA Pol II and CCNT1 occupancy across the proviral gene body, we used all Chip-Seq sequences attained and performed HIV-1 detection and assembly using the Burrows-Wheeler Aligner (BWA) v.0.7.17 [73]). To this end, we used the mem algorithm of BWA and the HXB2 HIV-1 reference genome (K03455.1). Subsequently, samtools v.1.17 was used to generate sorted.bam files and to calculate the depth of coverage with its *coverage* function. Coverage metrics where then analyzed and plotted using R v.4.3.2 in-house scripts and ggplot2 v.3.5.1 R package. In order to control for different inputs or sequencing depth that could bias viral quantification, we normalized the viral transcriptional quantification with equally bounded regions of the human genome under the different tested conditions. With this goal, we performed alignment, peak detection, and differential binding using the Chip-Seq human reads. To complete this analysis, we first performed sequencing data trimming using Trimmomatic v0.36 to remove adapters and low-quality reads. Trimmed reads were then aligned to the Homo sapiens reference genome GRCh38 using the Hisat2 v2.2.1 [74] disabling the splice alignment option. Subsequently, peaks were detected with MACS v3.0.1 [75] with a minimum FDR cutoff for peak detection of 0.01 using the input of each experiment as control. Finally, differential binding was tested using DiffBind v3.12.0 R package with the DESeq2 method. Two peaks corresponding with transcription start sites identified using ChIPpeakAnno v3.0.0 R package that were equally bound in the DMSO and KL2 conditions in the CCNT1 Chip-Seq or in the RNA Pol II ChIP-Seq [as determined by having some of the highest FDR values (FDR>0.99)] were selected as internal controls to normalize the HIV-1 reads. For the CCNT1 ChIP-Seq, we selected the *ALG6* transcription start site while for the RNA Pol II ChIP-Seq we used the *FNIP2* transcription start site. Reads quantified for these peaks by DiffBind were used as normalizing factors for the HIV-1 depth of coverage analysis.

### RNA sequencing

RNA samples from J-Lat 5A8 cells were submitted to Novogene for next-generation sequencing. Libraries were prepared using NEBNext Ultra II kit with poly(A) selection according to manufacturer protocols (New England Biolabs). Samples were sequenced on an Illumina NovaSeq X Plus with 150 bp paired-end reads.

### RNA sequencing analysis

Sequencing data was demultiplexed and trimmed using Trimmomatic v0.36 to remove adapters and low-quality reads. Trimmed reads were aligned to the Homo sapiens reference genome GRCh38 and transcripts quantified using the Hisat2-StringTie pipeline [76]. Differential gene expression analysis of the quantified gene transcripts was performed with DESeq2 v.1.42.0 R package using R v.4.3.2. After retaining genes with nonzero total read count and with more than 10 reads in total between all samples, we fitted a model that included all treatments to account for overall variability and identified differentially expressed genes (DEGs) within that model between all tested conditions against DMSO-treated cells (i.e. KL2 vs DMSO, AZD5582 vs DMSO, and AZD5582+KL2 vs DMSO). To define DEGs, we used as cut-offs an absolute log2 fold change > 1 and a false discovery rate (FDR) < 0.05 using the Benjamin-Hochberg procedure. Gene enrichment analyses for each comparison were subsequently performed using gene set enrichment analysis (GSEA) to identify specific Gene Ontologies (GO), KEGG, and REACTOME pathways associated with CO-iMs and/or HIV infection and ART treatment. We performed GSEA using clusterProfiler v.4.10.0 in R with all lists of genes ranked by the corresponding log2 fold change and compared the different treatments with the *compareCluster* function. For these analyses all genes whose gene symbols could be mapped to ENTREZ Ids using the org.Hs.eg.db v.3.18.0 Bioconductor annotation package were included.

### Tat-Rescue of U1 cells

Codon optimized HIV-1 NL4.3 Tat with a C-terminal 2xStrep(TagII)-TEV-3xFlag tag was ordered as a GeneBlock (IDT) and inserted into BamHI/ECORI linearized pLVX-TetOne-Puro by Gibson Assembly and subsequently sequence verified. Briefly, 10 μg of the molecular clone was transfected (PolyJet; SignaGen) into 5 × 10^6^ human embryonic kidney (HEK) 293T cells (ATCC, CRL-3216) according to the manufacturer’s protocol. Twenty-five mL of the supernatant was collected at 48 and 72 hours and then combined. The virus-containing supernatant was filtered through 0.45-mm polyvinylidene difluoride filters (Millipore) and precipitated in 8.5% polyethylene glycol [average molecular weight (Mn), 6000; Sigma-Aldrich] and 0.3 M NaCl for 4 hours at 4 °C. Supernatants were centrifuged at 3500 rpm for 20 minutes, and the virus was resuspended in 0.25 ml of PBS for a 100X effective concentration. Aliquots were stored at −80 °C until use. Aliquots were thawed and added to cultures of U1 cells at a concentration of 1:100 in complete RPMI and cells were cultured for 48 hours. Following transduction, the cells were pelleted, and media was removed and replaced with fresh complete RPMI + 10 μg/mL of puromycin to select for successfully transduced cells. Cells were cultured in selective growth media for 7 days. After selection, cells were plated in 96-well flat-bottom plates according to previous methods and treated with latency reversing agents and doxycycline for 48 hours before analysis of p24 expressing cells by flow cytometry.

### Treatment of patient PBMCs

#### Study subjects

We selected five study subjects enrolled in the Northwestern University Clinical Research Site for the MWCCS who were well-suppressed [undetectable plasma HIV-1 (<50 copies/ml)] and had received antiretroviral drugs for at least 5 years. We obtained patient PBMCs from cryostorage. Laboratory procedures for clinical sample management are described previously [77]. The Institutional Review Board of Northwestern University approved the study (STU00022906-CR0008) with most recent approval date of May 16, 2022. All participants provided written informed consent.

#### Total RNA isolation

Total RNA was isolated from cultures of 1 x 10^6^ PBMCs that were treated with for 48 hours with JQ1, or AZD5582 in the presence or absence of KL-2 using an RNeasy kit (Qiagen), with the optional on-column deoxyribonuclease I digestion step. The isolated total RNA was eluted in ribonuclease-free water, cleaned, and concentrated using the RNA Clean and Concentration kit (Zymo) and assessed for quantity and quality by Qubit (Thermo Fisher Scientific) and 4200 TapeStation (Agilent), respectively before qRT-PCR.

#### Validation of cell-associated HIV-1 RNA

We performed real-time qRT-PCR using an HIV-1-gag–specific primers-probe (FAM) set: HIV-1-gagF, 5′-GGTGCGAGAGCGTCAGTATTAAG-3′; HIV-1-gagR, 5′-AGCTCCCTGCTTGCCCATA-3′; HIV-1-gagProbe, 6FAM-5′-TGGGAAAAAATTCGGTTAAGGCCAGGG-3′-QSY. We used the lactate dehydrogenase A (LDHA) gene for the internal normalization primers-probe (VIC) set (VIC-MGB: assay ID Hs03405707_g1; TaqMan Gene Expression Assay, Thermo Fisher Scientific). Briefly, a 10-μL RT-PCR mixture contained TaqMan Fast Virus 1-Step Master Mix, 400 nM forward and reverse HIV-1-gag primers, 0.3 μL of LDHA Gene Expression Assay (Thermo Fisher Scientific), 250 nM each of the probes, and 5 μL of extracted RNA or water for the no template controls. We programmed the 7900HT real-time PCR system (Applied Biosystems) for 20 minutes at 50 °C and 20 seconds at 95 °C, followed by 40 cycles of 15 seconds at 95 °C and 60 seconds at 60 °C. The qRT-PCR data were analyzed in technical triplicate. We calculated the fold change in gene expression using the standard 2^-ΔΔCT method.

### Statistical analysis

All statistical analysis was performed using GraphPad Prism version 10.2.0 (392) for Windows 64-bit, GraphPad Software, Boston, Massachusetts, USA (www.graphpad.com).

## Supporting information

S1 Fig Primary cell viability and gating strategy. **A)** Percent viable primary CD4+ T cells (normalized to the donor-matched NT control) 72 hours after electroporation with multiplexed CRISPR-Cas9 RNPs targeting the indicated genes as measured by amine dye staining and flow cytometry. Each dot represents the average of technical triplicates; the black line represents the mean of means ± standard error. n = 12 donors for NT, CXCR4, CCNT1, CDK9, AFF1, AFF4, and ELL2; n = 6 donors for AF9; n = 9 donors for ENL. Statistics were calculated by two-way ANOVA with Dunnet’s Multiple Comparison Test; no significant differences were observed. **B)** Gating strategy for quantification of percent viable primary CD4+ T cells via sequential application of a live cell gate, two single-cell gates, and a fluorophore gate (FlowJo v10.7.1). **C)** Gating strategy for quantification of percent HIV-1 infection in primary CD4+ T cells via sequential application of a live cell gate, two single-cell gates, and autofluorescence exclusion (FlowJo v10.7.1).
(TIF)

S2 Fig Histograms of CD4 and CXCR4 expression on primary CD4+ T cells after KL-2 treatment. **A)** Histogram of cell surface CD4 expression on activated CD4+ T cells treated with DMSO or 3.125 μM KL-2 for 48 hours as measured by immunostaining and flow cytometry (one representative donor, visualized in FlowJo v10.7.1). **B)** Histogram of cell surface CXCR4 expression on activated CD4+ T cells treated with DMSO or 3.125 μM KL-2 for 48 hours as measured by immunostaining and flow cytometry (one representative donor, visualized in FlowJo v10.7.1). **C)** Luminescence of lysed CHME3 cells (normalized to the DMSO control) 48 hours after challenge with HIV-1 NL4.3 dNef:NanoLuc in the presence of increasing concentrations of KL-2 (24 hours pre-treatment before challenge). Data represent the average ± standard deviation of technical triplicates (n = 3 donors); statistics were calculated relative to the DMSO control by two-way ANOVA and Sidak’s Multiple Comparison test with significant p-values (p < 0.05) shown. **D)** Viablity as monitored by CellTiter-Glo of CHME3 cells (normalized to the DMSO control) after 48 hours of treatment with increasing concentrations of KL-2. Data represent the average ± standard deviation of technical triplicates.
(TIF)

S3 Fig J-Lat cell viability upon LRA treatment and reactivation. **A)** Percent viable J-Lat 5A8 cells (normalized to the DMSO-treated control) 48 hours after treatment with the indicated LRAs in the presence and absence of 6.25 μM KL-2 as measured by amine dye staining and flow cytometry. Each bar represents the average ± standard deviation of technical triplicates. The same data are shown for **B)** J-Lat 6.3 cells and **C)** J-Lat 11.1 cells. **D)** Excess over Bliss scores for latency reactivation in J-Lat 5A8 cells treated with combinations of KL-2 and AZD5582 (top) or JQ1 (bottom). Cells were treated in biological duplicates with the mean excess over Bliss score +/- standard deviation between replicates shown.
(TIF)

S4 Fig J-Lat, U1, and ACH-2 cell viability upon LRA treatment and reactivation. **A)** Percent viable U1 cells 48 hours after treatment with the indicated LRAs in the presence and absence of 6.25 μM KL-2 as measured by amine dye staining and flow cytometry. Each bar represents the average ± standard deviation of technical triplicates. **B)** Percent viable ACH-2 cells 48 hours after treatment with the indicated LRAs in the presence and absence of 6.25 μM KL-2 as measured by amine dye staining and flow cytometry. Each bar represents the average ± standard deviation of technical triplicates. **C)** Percent reactivated (KC57-FITC+) Lenti-Tat U1 cells after 48 hours of treatment with increasing concentrations of doxycycline. **D)** Percent viable Lenti-Tat U1 cells 48 hours after treatment with the indicated LRAs in the presence and absence of 6.25 μM KL-2 and differing amounts of doxycycline as measured by amine dye staining and flow cytometry. Each bar represents the average ± standard deviation of technical triplicates. **E)** Immunoblotting of whole cell lysates from the indicate cell lines using primary antibodies against AFF4, CCNT1, CDK9, and β-Actin. **F)** Percent viable J-Lat 5A8 cells 48 hours after treatment with the indicated LRAs in the presence and absence of 6.25 μM KL-2 and the Tat inhibitors Spironolactone and Triptolide as measured by amine dye staining and flow cytometry. Each bar represents the average ± standard deviation of technical triplicates. **G)** Percent reactivated (GFP+) J-Lat A2 cells (normalized to the DMSO control) after 48 hours of treatment with increasing concentrations of KL-2. Data represent the average ± standard deviation of technical triplicates; statistics were calculated relative to the DMSO control by two-way ANOVA and Sidak’s Multiple Comparison test. **H)** Percent reactivated (GFP+) J-Lat A72 cells (normalized to the DMSO control) after 48 hours of treatment with increasing concentrations of KL-2. Data represent the average ± standard deviation of technical triplicates; statistics were calculated relative to the DMSO control by two-way ANOVA and Sidak’s Multiple Comparison test.
(TIF)

## References

[1] Siliciano RF, Greene WC. HIV latency. Cold Spring Harb Perspect Med. 2011;1(1):a007096. doi: 10.1101/cshperspect.a007096 22229121 PMC3234450

[2] Margolis DM, Archin NM, Cohen MS, Eron JJ, Ferrari G, Garcia JV, et al. Curing HIV: Seeking to Target and Clear Persistent Infection. Cell. 2020;181(1):189–206. doi: 10.1016/j.cell.2020.03.005 32220311 PMC7896558

[3] Li JZ, Aga E, Bosch RJ, Pilkinton M, Kroon E, MacLaren L, et al. Time to Viral Rebound After Interruption of Modern Antiretroviral Therapies. Clin Infect Dis. 2022;74(5):865–70. doi: 10.1093/cid/ciab541 34117753 PMC8906742

[4] Colven R, Harrington RD, Spach DH, Cohen CJ, Hooton TM. Retroviral rebound syndrome after cessation of suppressive antiretroviral therapy in three patients with chronic HIV infection. Ann Intern Med. 2000;133(6):430–4. doi: 10.7326/0003-4819-133-6-200009190-00010 10975960

[5] Min S, Gillani FS, Aung S, Garland JM, Beckwith CG. Evaluating HIV Viral Rebound Among Persons on Suppressive Antiretroviral Treatment in the Era of "Undetectable Equals Untransmittable (U = U)". Open Forum Infect Dis. 2020;7(12):ofaa529. 33335935 10.1093/ofid/ofaa529 PMC7731526

[6] Kim Y, Anderson JL, Lewin SR. Getting the "Kill" into "Shock and Kill": Strategies to Eliminate Latent HIV. Cell Host Microbe. 2018;23(1):14–26. doi: 10.1016/j.chom.2017.12.004 29324227 PMC5990418

[7] Vansant G, Bruggemans A, Janssens J, Debyser Z. Block-And-Lock Strategies to Cure HIV Infection. Viruses. 2020;12(1). doi: 10.3390/v12010084 31936859 PMC7019976

[8] Singh V, Dashti A, Mavigner M, Chahroudi A. Latency Reversal 2.0: Giving the Immune System a Seat at the Table. Curr HIV/AIDS Rep. 2021;18(2):117–27. doi: 10.1007/s11904-020-00540-z 33433817 PMC7985101

[9] Spivak AM, Planelles V. Novel Latency Reversal Agents for HIV-1 Cure. Annu Rev Med. 2018;69:421–36. doi: 10.1146/annurev-med-052716-031710 29099677 PMC5892446

[10] Cillo AR, Sobolewski MD, Bosch RJ, Fyne E, Piatak M Jr., Coffin JM, et al. Quantification of HIV-1 latency reversal in resting CD4+ T cells from patients on suppressive antiretroviral therapy. Proc Natl Acad Sci U S A. 2014;111(19):7078–83. doi: 10.1073/pnas.1402873111 24706775 PMC4024870

[11] Rodari A, Darcis G, Van Lint CM. The Current Status of Latency Reversing Agents for HIV-1 Remission. Annu Rev Virol. 2021;8(1):491–514. doi: 10.1146/annurev-virology-091919-103029 34586875

[12] Falcinelli SD, Peterson JJ, Turner AW, Irlbeck D, Read J, Raines SL, et al. Combined noncanonical NF-κB agonism and targeted BET bromodomain inhibition reverse HIV latency ex vivo. J Clin Invest. 2022;132(8). 10.1172/JCI157281 PMC9012286 35426377

[13] Yukl SA, Kaiser P, Kim P, Telwatte S, Joshi SK, Vu M, et al. HIV latency in isolated patient CD4(+) T cells may be due to blocks in HIV transcriptional elongation, completion, and splicing. Sci Transl Med. 2018;10(430). doi: 10.1126/scitranslmed.aap9927 29491188 PMC5959841

[14] Horvath RM, Dahabieh M, Malcolm T, Sadowski I. TRIM24 controls induction of latent HIV-1 by stimulating transcriptional elongation. Commun Biol. 2023;6(1):86. doi: 10.1038/s42003-023-04484-z 36690785 PMC9870992

[15] Adelman K, Lis JT. Promoter-proximal pausing of RNA polymerase II: emerging roles in metazoans. Nat Rev Genet. 2012;13(10):720–31. doi: 10.1038/nrg3293 22986266 PMC3552498

[16] Soliman SHA, Cisneros WJ, Iwanaszko M, Aoi Y, Ganesan S, Walter M, et al. Enhancing HIV-1 latency reversal through regulating the elongating RNA Pol II pause-release by a small-molecule disruptor of PAF1C. Sci Adv. 2023;9(10):eadf2468. doi: 10.1126/sciadv.adf2468 36888719 PMC9995073

[17] Tantale K, Garcia-Oliver E, Robert MC, L’Hostis A, Yang Y, Tsanov N, et al. Stochastic pausing at latent HIV-1 promoters generates transcriptional bursting. Nat Commun. 2021;12(1):4503. doi: 10.1038/s41467-021-24462-5 34301927 PMC8302722

[18] Gao R, Bao J, Yan H, Xie L, Qin W, Ning H, et al. Competition between PAF1 and MLL1/COMPASS confers the opposing function of LEDGF/p75 in HIV latency and proviral reactivation. Sci Adv. 2020;6(20):eaaz8411. doi: 10.1126/sciadv.aaz8411 32426500 PMC7220354

[19] Marshall NF, Price DH. Purification of P-TEFb, a transcription factor required for the transition into productive elongation. J Biol Chem. 1995;270(21):12335–8. doi: 10.1074/jbc.270.21.12335 7759473

[20] Marshall NF, Price DH. Control of formation of two distinct classes of RNA polymerase II elongation complexes. Mol Cell Biol. 1992;12(5):2078–90. doi: 10.1128/mcb.12.5.2078-2090.1992 1569941 PMC364379

[21] Peng J, Zhu Y, Milton JT, Price DH. Identification of multiple cyclin subunits of human P-TEFb. Genes Dev. 1998;12(5):755–62. doi: 10.1101/gad.12.5.755 9499409 PMC316581

[22] Hsin JP, Manley JL. The RNA polymerase II CTD coordinates transcription and RNA processing. Genes Dev. 2012;26(19):2119–37. doi: 10.1101/gad.200303.112 23028141 PMC3465734

[23] Price DH. P-TEFb, a cyclin-dependent kinase controlling elongation by RNA polymerase II. Mol Cell Biol. 2000;20(8):2629–34. doi: 10.1128/MCB.20.8.2629-2634.2000 10733565 PMC85478

[24] Peterlin BM, Price DH. Controlling the elongation phase of transcription with P-TEFb. Mol Cell. 2006;23(3):297–305. doi: 10.1016/j.molcel.2006.06.014 16885020

[25] Chen R, Yang Z, Zhou Q. Phosphorylated positive transcription elongation factor b (P-TEFb) is tagged for inhibition through association with 7SK snRNA. J Biol Chem. 2004;279(6):4153–60. doi: 10.1074/jbc.M310044200 14627702

[26] Yik JH, Chen R, Nishimura R, Jennings JL, Link AJ, Zhou Q. Inhibition of P-TEFb (CDK9/Cyclin T) kinase and RNA polymerase II transcription by the coordinated actions of HEXIM1 and 7SK snRNA. Mol Cell. 2003;12(4):971–82. doi: 10.1016/s1097-2765(03)00388-5 14580347

[27] Krueger BJ, Varzavand K, Cooper JJ, Price DH. The mechanism of release of P-TEFb and HEXIM1 from the 7SK snRNP by viral and cellular activators includes a conformational change in 7SK. PLoS One. 2010;5(8):e12335. doi: 10.1371/journal.pone.0012335 20808803 PMC2925947

[28] Chen R, Liu M, Li H, Xue Y, Ramey WN, He N, et al. PP2B and PP1alpha cooperatively disrupt 7SK snRNP to release P-TEFb for transcription in response to Ca2+ signaling. Genes Dev. 2008;22(10):1356–68. doi: 10.1101/gad.1636008 18483222 PMC2377190

[29] Bartholomeeusen K, Xiang Y, Fujinaga K, Peterlin BM. Bromodomain and extra-terminal (BET) bromodomain inhibition activate transcription via transient release of positive transcription elongation factor b (P-TEFb) from 7SK small nuclear ribonucleoprotein. J Biol Chem. 2012;287(43):36609–16. doi: 10.1074/jbc.M112.410746 22952229 PMC3476326

[30] Barboric M, Nissen RM, Kanazawa S, Jabrane-Ferrat N, Peterlin BM. NF-kappaB binds P-TEFb to stimulate transcriptional elongation by RNA polymerase II. Mol Cell. 2001;8(2):327–37. doi: 10.1016/s1097-2765(01)00314-8 11545735

[31] Eberhardy SR, Farnham PJ. Myc recruits P-TEFb to mediate the final step in the transcriptional activation of the cad promoter. J Biol Chem. 2002;277(42):40156–62. doi: 10.1074/jbc.M207441200 12177005

[32] Liang K, Smith ER, Aoi Y, Stoltz KL, Katagi H, Woodfin AR, et al. Targeting Processive Transcription Elongation via SEC Disruption for MYC-Induced Cancer Therapy. Cell. 2018;175(3):766–79.e17. doi: 10.1016/j.cell.2018.09.027 30340042 PMC6422358

[33] Yang Z, Yik JH, Chen R, He N, Jang MK, Ozato K, et al. Recruitment of P-TEFb for stimulation of transcriptional elongation by the bromodomain protein Brd4. Mol Cell. 2005;19(4):535–45. doi: 10.1016/j.molcel.2005.06.029 16109377

[34] Itzen F, Greifenberg AK, Bösken CA, Geyer M. Brd4 activates P-TEFb for RNA polymerase II CTD phosphorylation. Nucleic Acids Res. 2014;42(12):7577–90. doi: 10.1093/nar/gku449 24860166 PMC4081074

[35] Luo Z, Lin C, Guest E, Garrett AS, Mohaghegh N, Swanson S, et al. The super elongation complex family of RNA polymerase II elongation factors: gene target specificity and transcriptional output. Mol Cell Biol. 2012;32(13):2608–17. doi: 10.1128/MCB.00182-12 22547686 PMC3434493

[36] Tahirov TH, Babayeva ND, Varzavand K, Cooper JJ, Sedore SC, Price DH. Crystal structure of HIV-1 Tat complexed with human P-TEFb. Nature. 2010;465(7299):747–51. doi: 10.1038/nature09131 20535204 PMC2885016

[37] Zhu Y, Pe’ery T, Peng J, Ramanathan Y, Marshall N, Marshall T, et al. Transcription elongation factor P-TEFb is required for HIV-1 tat transactivation in vitro. Genes Dev. 1997;11(20):2622–32. doi: 10.1101/gad.11.20.2622 9334325 PMC316609

[38] Molle D, Maiuri P, Boireau S, Bertrand E, Knezevich A, Marcello A, et al. A real-time view of the TAR:Tat:P-TEFb complex at HIV-1 transcription sites. Retrovirology. 2007;4:36. doi: 10.1186/1742-4690-4-36 17537237 PMC1904240

[39] Stoszko M, Al-Hatmi AMS, Skriba A, Roling M, Ne E, Crespo R, et al. Gliotoxin, identified from a screen of fungal metabolites, disrupts 7SK snRNP, releases P-TEFb, and reverses HIV-1 latency. Sci Adv. 2020;6(33):eaba6617. doi: 10.1126/sciadv.aba6617 32851167 PMC7423394

[40] Mbonye UR, Gokulrangan G, Datt M, Dobrowolski C, Cooper M, Chance MR, et al. Phosphorylation of CDK9 at Ser175 enhances HIV transcription and is a marker of activated P-TEFb in CD4(+) T lymphocytes. PLoS Pathog. 2013;9(5):e1003338. doi: 10.1371/journal.ppat.1003338 23658523 PMC3642088

[41] Chou S, Upton H, Bao K, Schulze-Gahmen U, Samelson AJ, He N, et al. HIV-1 Tat recruits transcription elongation factors dispersed along a flexible AFF4 scaffold. Proc Natl Acad Sci U S A. 2013;110(2):E123–31. doi: 10.1073/pnas.1216971110 23251033 PMC3545800

[42] Schulze-Gahmen U, Upton H, Birnberg A, Bao K, Chou S, Krogan NJ, et al. The AFF4 scaffold binds human P-TEFb adjacent to HIV Tat. Elife. 2013;2:e00327. doi: 10.7554/eLife.00327 23471103 PMC3589825

[43] Hultquist JF, Hiatt J, Schumann K, McGregor MJ, Roth TL, Haas P, et al. CRISPR-Cas9 genome engineering of primary CD4(+) T cells for the interrogation of HIV-host factor interactions. Nat Protoc. 2019;14(1):1–27. doi: 10.1038/s41596-018-0069-7 30559373 PMC6637941

[44] Hultquist JF, Schumann K, Woo JM, Manganaro L, McGregor MJ, Doudna J, et al. A Cas9 Ribonucleoprotein Platform for Functional Genetic Studies of HIV-Host Interactions in Primary Human T Cells. Cell Rep. 2016;17(5):1438–52. doi: 10.1016/j.celrep.2016.09.080 27783955 PMC5123761

[45] Hiatt J, Hultquist JF, McGregor MJ, Bouhaddou M, Leenay RT, Simons LM, et al. A functional map of HIV-host interactions in primary human T cells. Nat Commun. 2022;13(1):1752. doi: 10.1038/s41467-022-29346-w 35365639 PMC8976027

[46] Wang D, Yin Z, Wang H, Wang L, Li T, Xiao R, et al. The super elongation complex drives transcriptional addiction in MYCN-amplified neuroblastoma. Sci Adv. 2023;9(13):eadf0005. doi: 10.1126/sciadv.adf0005 36989355 PMC10058231

[47] Gu F, Boisjoli M, Naghavi MH. HIV-1 promotes ubiquitination of the amyloidogenic C-terminal fragment of APP to support viral replication. Nat Commun. 2023;14(1):4227. doi: 10.1038/s41467-023-40000-x 37454116 PMC10349857

[48] Li Z, Guo J, Wu Y, Zhou Q. The BET bromodomain inhibitor JQ1 activates HIV latency through antagonizing Brd4 inhibition of Tat-transactivation. Nucleic Acids Res. 2013;41(1):277–87. doi: 10.1093/nar/gks976 23087374 PMC3592394

[49] Chan JK, Bhattacharyya D, Lassen KG, Ruelas D, Greene WC. Calcium/calcineurin synergizes with prostratin to promote NF-κB dependent activation of latent HIV. PLoS One. 2013;8(10):e77749. 24204950 10.1371/journal.pone.0077749 PMC3813743

[50] Liu Q, Yin X, Languino LR, Altieri DC. Evaluation of drug combination effect using a Bliss independence dose-response surface model. Stat Biopharm Res. 2018;10(2):112–22. doi: 10.1080/19466315.2018.1437071 30881603 PMC6415926

[51] Chen FX, Smith ER, Shilatifard A. Born to run: control of transcription elongation by RNA polymerase II. Nature Reviews Molecular Cell Biology. 2018;19(7):464–78. doi: 10.1038/s41580-018-0010-5 29740129

[52] Emiliani S, Fischle W, Ott M, Van Lint C, Amella CA, Verdin E. Mutations in the tat gene are responsible for human immunodeficiency virus type 1 postintegration latency in the U1 cell line. J Virol. 1998;72(2):1666–70. doi: 10.1128/JVI.72.2.1666-1670.1998 9445075 PMC124653

[53] Emiliani S, Van Lint C, Fischle W, Paras P Jr., Ott M, Brady J, et al. A point mutation in the HIV-1 Tat responsive element is associated with postintegration latency. Proc Natl Acad Sci U S A. 1996;93(13):6377–81. doi: 10.1073/pnas.93.13.6377 8692823 PMC39030

[54] Lacombe B, Morel M, Margottin-Goguet F, Ramirez BC. Specific Inhibition of HIV Infection by the Action of Spironolactone in T Cells. J Virol. 2016;90(23):10972–80. doi: 10.1128/JVI.01722-16 27681137 PMC5110165

[55] Wan Z, Chen X. Triptolide inhibits human immunodeficiency virus type 1 replication by promoting proteasomal degradation of Tat protein. Retrovirology. 2014;11:88. doi: 10.1186/s12977-014-0088-6 25323821 PMC4205289

[56] Wang Y, Lu JJ, He L, Yu Q. Triptolide (TPL) inhibits global transcription by inducing proteasome-dependent degradation of RNA polymerase II (Pol II). PLoS One. 2011;6(9):e23993. doi: 10.1371/journal.pone.0023993 21931633 PMC3172214

[57] Mori L, Jenike K, Yeh YJ, Lacombe B, Li C, Getzler A, et al. The XPB Subunit of the TFIIH Complex Plays a Critical Role in HIV-1 Transcription and XPB Inhibition by Spironolactone Prevents HIV-1 Reactivation from Latency. J Virol. 2021;95(4). 10.1128/JVI.01247-20 PMC7851559 33239456

[58] Yang J, Tang X, Ke X, Dai Y, Shi J. Triptolide Suppresses NF-κB-Mediated Inflammatory Responses and Activates Expression of Nrf2-Mediated Antioxidant Genes to Alleviate Caerulein-Induced Acute Pancreatitis. Int J Mol Sci. 2022;23(3). 10.3390/ijms23031252 PMC8835869 35163177

[59] Tian Q, Zhang P, Wang Y, Si Y, Yin D, Weber CR, et al. A novel triptolide analog downregulates NF-κB and induces mitochondrial apoptosis pathways in human pancreatic cancer. eLife. 2023;12:e85862. 37877568 10.7554/eLife.85862 PMC10861173

[60] Mediouni S, Chinthalapudi K, Ekka MK, Usui I, Jablonski JA, Clementz MA, et al. Didehydro-Cortistatin A Inhibits HIV-1 by Specifically Binding to the Unstructured Basic Region of Tat. mBio. 2019;10(1). 10.1128/mBio.02662-18 PMC6368365 30723126

[61] Jordan A, Defechereux P, Verdin E. The site of HIV-1 integration in the human genome determines basal transcriptional activity and response to Tat transactivation. Embo j. 2001;20(7):1726–38. doi: 10.1093/emboj/20.7.1726 11285236 PMC145503

[62] Jordan A, Bisgrove D, Verdin E. HIV reproducibly establishes a latent infection after acute infection of T cells in vitro. Embo j. 2003;22(8):1868–77. doi: 10.1093/emboj/cdg188 12682019 PMC154479

[63] Zhu J, Gaiha GD, John SP, Pertel T, Chin CR, Gao G, et al. Reactivation of latent HIV-1 by inhibition of BRD4. Cell Rep. 2012;2(4):807–16. doi: 10.1016/j.celrep.2012.09.008 23041316 PMC3523124

[64] Kuzmina A, Krasnopolsky S, Taube R. Super elongation complex promotes early HIV transcription and its function is modulated by P-TEFb. Transcription. 2017;8(3):133–49. doi: 10.1080/21541264.2017.1295831 28340332 PMC5501376

[65] He N, Liu M, Hsu J, Xue Y, Chou S, Burlingame A, et al. HIV-1 Tat and host AFF4 recruit two transcription elongation factors into a bifunctional complex for coordinated activation of HIV-1 transcription. Mol Cell. 2010;38(3):428–38. doi: 10.1016/j.molcel.2010.04.013 20471948 PMC3085314

[66] Alamer E, Zhong C, Hajnik R, Soong L, Hu H. Modulation of BRD4 in HIV epigenetic regulation: implications for finding an HIV cure. Retrovirology. 2021;18(1):3. doi: 10.1186/s12977-020-00547-9 33413475 PMC7792063

[67] Gressel S, Schwalb B, Decker TM, Qin W, Leonhardt H, Eick D, et al. CDK9-dependent RNA polymerase II pausing controls transcription initiation. Elife. 2017;6. doi: 10.7554/eLife.29736 28994650 PMC5669633

[68] Nekhai S, Petukhov M, Breuer D. Regulation of CDK9 activity by phosphorylation and dephosphorylation. Biomed Res Int. 2014;2014:964964. doi: 10.1155/2014/964964 24524087 PMC3913462

[69] Ammosova T, Obukhov Y, Kotelkin A, Breuer D, Beullens M, Gordeuk VR, et al. Protein phosphatase-1 activates CDK9 by dephosphorylating Ser175. PLoS One. 2011;6(4):e18985. doi: 10.1371/journal.pone.0018985 21533037 PMC3080879

[70] Anshabo AT, Milne R, Wang S, Albrecht H. CDK9: A Comprehensive Review of Its Biology, and Its Role as a Potential Target for Anti-Cancer Agents. Front Oncol. 2021;11:678559. doi: 10.3389/fonc.2021.678559 34041038 PMC8143439

[71] Titov DV, Gilman B, He QL, Bhat S, Low WK, Dang Y, et al. XPB, a subunit of TFIIH, is a target of the natural product triptolide. Nat Chem Biol. 2011;7(3):182–8. doi: 10.1038/nchembio.522 21278739 PMC3622543

[72] Lee TI, Johnstone SE, Young RA. Chromatin immunoprecipitation and microarray-based analysis of protein location. Nat Protoc. 2006;1(2):729–48. doi: 10.1038/nprot.2006.98 17406303 PMC3004291

[73] Li H, Durbin R. Fast and accurate short read alignment with Burrows-Wheeler transform. Bioinformatics. 2009;25(14):1754–60. doi: 10.1093/bioinformatics/btp324 19451168 PMC2705234

[74] Kim D, Paggi JM, Park C, Bennett C, Salzberg SL. Graph-based genome alignment and genotyping with HISAT2 and HISAT-genotype. Nat Biotechnol. 2019;37(8):907–915. doi: 10.1038/s41587-019-0201-4 31375807 PMC7605509

[75] Zhang Y, Liu T, Meyer CA, Eeckhoute J, Johnson DS, Bernstein BE, Nusbaum C, Myers RM, Brown M, Li W, Liu XS. Model-based analysis of ChIP-Seq (MACS). Genome Biol. 2008;9(9):R137. doi: 10.1186/gb-2008-9-9-r137 18798982 PMC2592715

[76] Pertea M, Kim D, Pertea GM, Leek JT, Salzberg SL. Transcript-level expression analysis of RNA-seq experiments with HISAT, StringTie and Ballgown. Nat Protoc. 2016;11(9):1650–67. doi: 10.1038/nprot.2016.095 27560171 PMC5032908

[77] Detels R, Jacobson L, Margolick J, Martinez-Maza O, Muñoz A, Phair J, et al. The multicenter AIDS Cohort Study, 1983 to …. Public Health. 2012;126(3):196–8. doi: 10.1016/j.puhe.2011.11.013 22206985 PMC3324261

