# Supplementary figures and images for: Release of P-TEFb from the Super Elongation Complex promotes HIV-1 latency reversal
Source: PLoS Pathog. 2024 Sep 11;20(9):e1012083. doi: 10.1371/journal.ppat.1012083 (PMC11419360; doi:10.1371/journal.ppat.1012083)

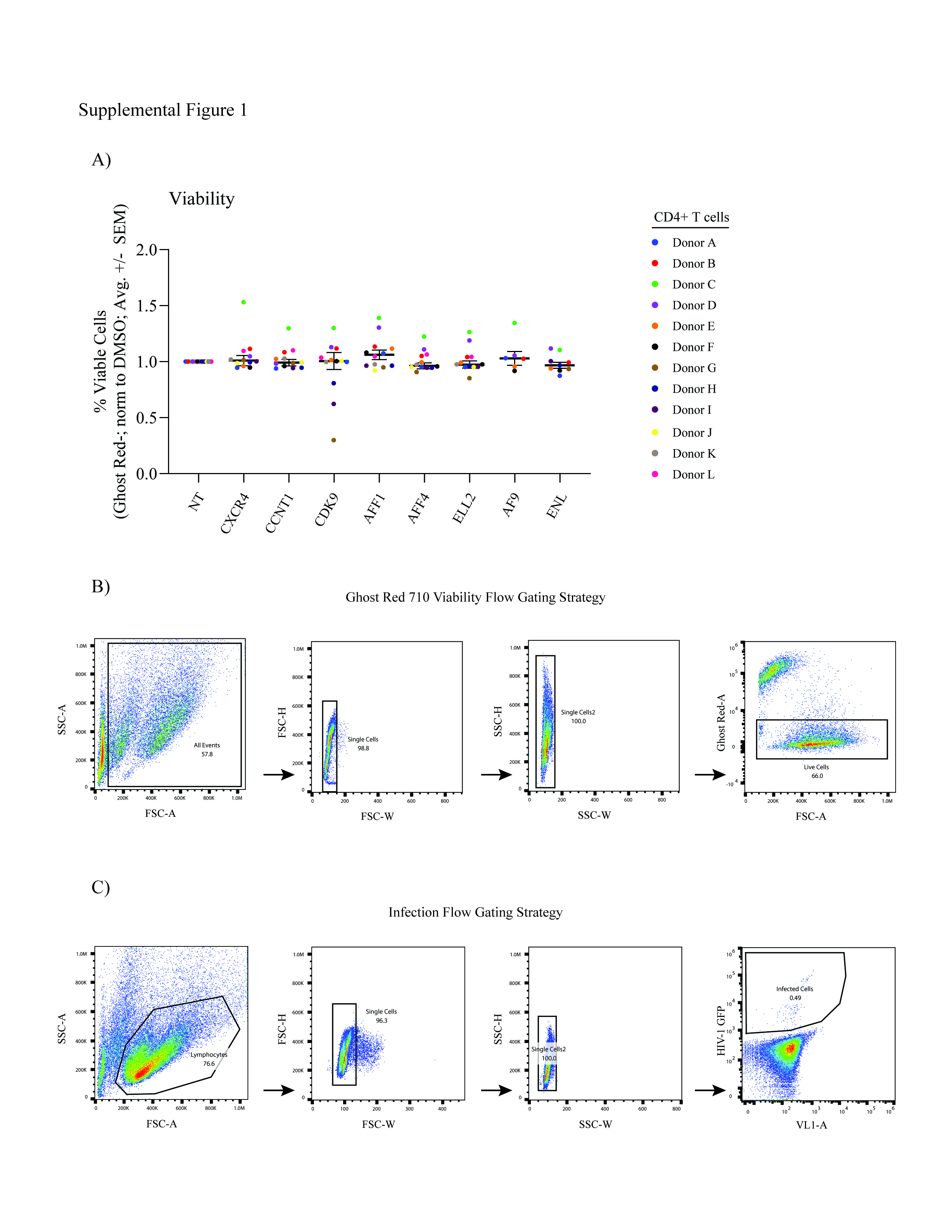

Supplement: S1 Fig — A) Percent viable primary CD4+ T cells (normalized to the donor-matched NT control) 72 hours after electroporation with multiplexed CRISPR-Cas9 RNPs targeting the indicated genes as measured by amine dye staining and flow cytometry. Each dot represents the average of technical triplicates; the black line represents the mean of means ± standard error. n = 12 donors for NT, CXCR4, CCNT1, CDK9, AFF1, AFF4, and ELL2; n = 6 donors for AF9; n = 9 donors for ENL. Statistics were calculated by two-way ANOVA with Dunnet’s Multiple Comparison Test; no significant differences were observed. B) Gating strategy for quantification of percent viable primary CD4+ T cells via sequential application of a live cell gate, two single-cell gates, and a fluorophore gate (FlowJo v10.7.1). C) Gating strategy for quantification of percent HIV-1 infection in primary CD4+ T cells via sequential application of a live cell gate, two single-cell gates, and autofluorescence exclusion (FlowJo v10.7.1). (TIF) [file ppat.1012083.s001.tif]

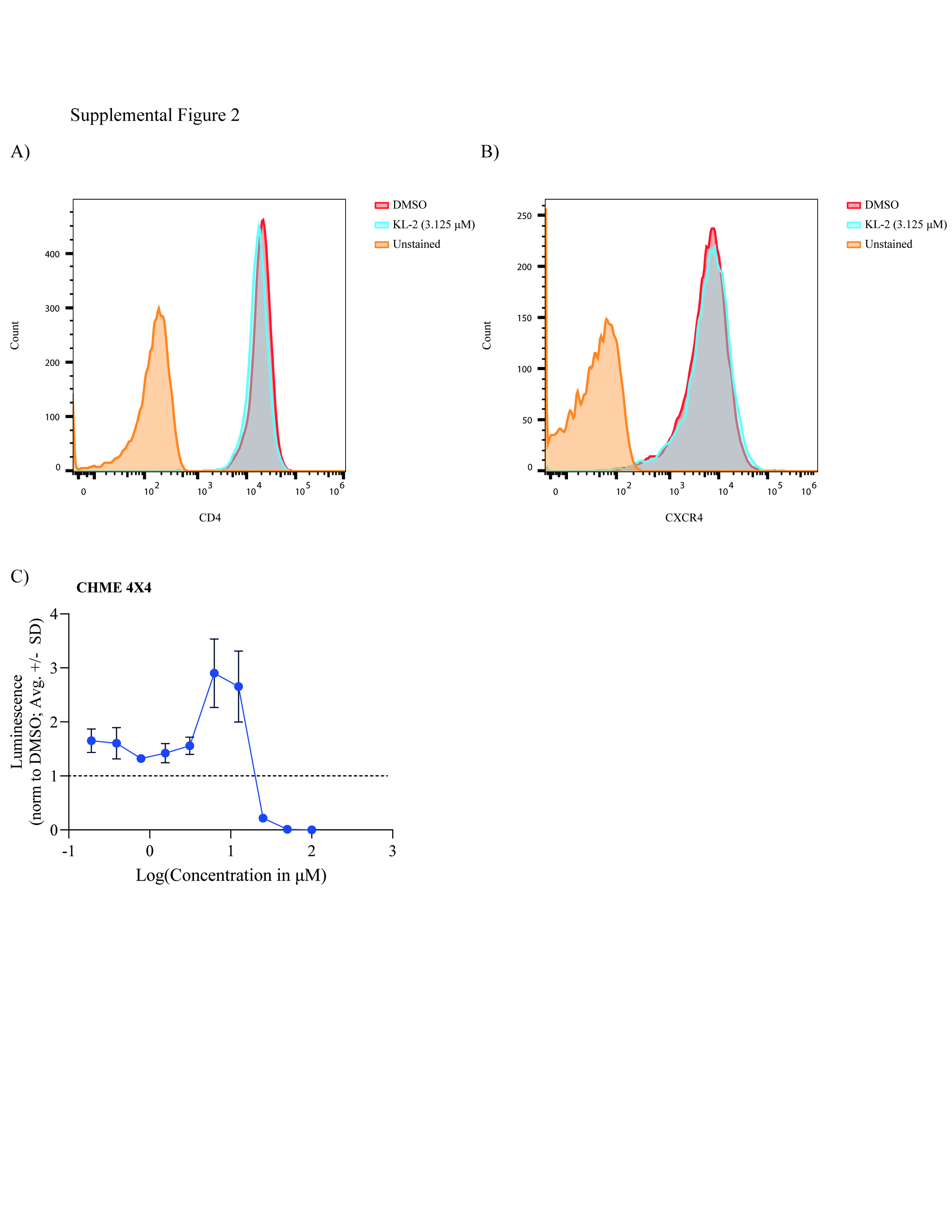

Supplement: S2 Fig — A) Histogram of cell surface CD4 expression on activated CD4+ T cells treated with DMSO or 3.125 μM KL-2 for 48 hours as measured by immunostaining and flow cytometry (one representative donor, visualized in FlowJo v10.7.1). B) Histogram of cell surface CXCR4 expression on activated CD4+ T cells treated with DMSO or 3.125 μM KL-2 for 48 hours as measured by immunostaining and flow cytometry (one representative donor, visualized in FlowJo v10.7.1). C) Luminescence of lysed CHME3 cells (normalized to the DMSO control) 48 hours after challenge with HIV-1 NL4.3 dNef:NanoLuc in the presence of increasing concentrations of KL-2 (24 hours pre-treatment before challenge). Data represent the average ± standard deviation of technical triplicates (n = 3 donors); statistics were calculated relative to the DMSO control by two-way ANOVA and Sidak’s Multiple Comparison test with significant p-values (p < 0.05) shown. D) Viablity as monitored by CellTiter-Glo of CHME3 cells (normalized to the DMSO control) after 48 hours of treatment with increasing concentrations of KL-2. Data represent the average ± standard deviation of technical triplicates. (TIF) [file ppat.1012083.s002.tif]

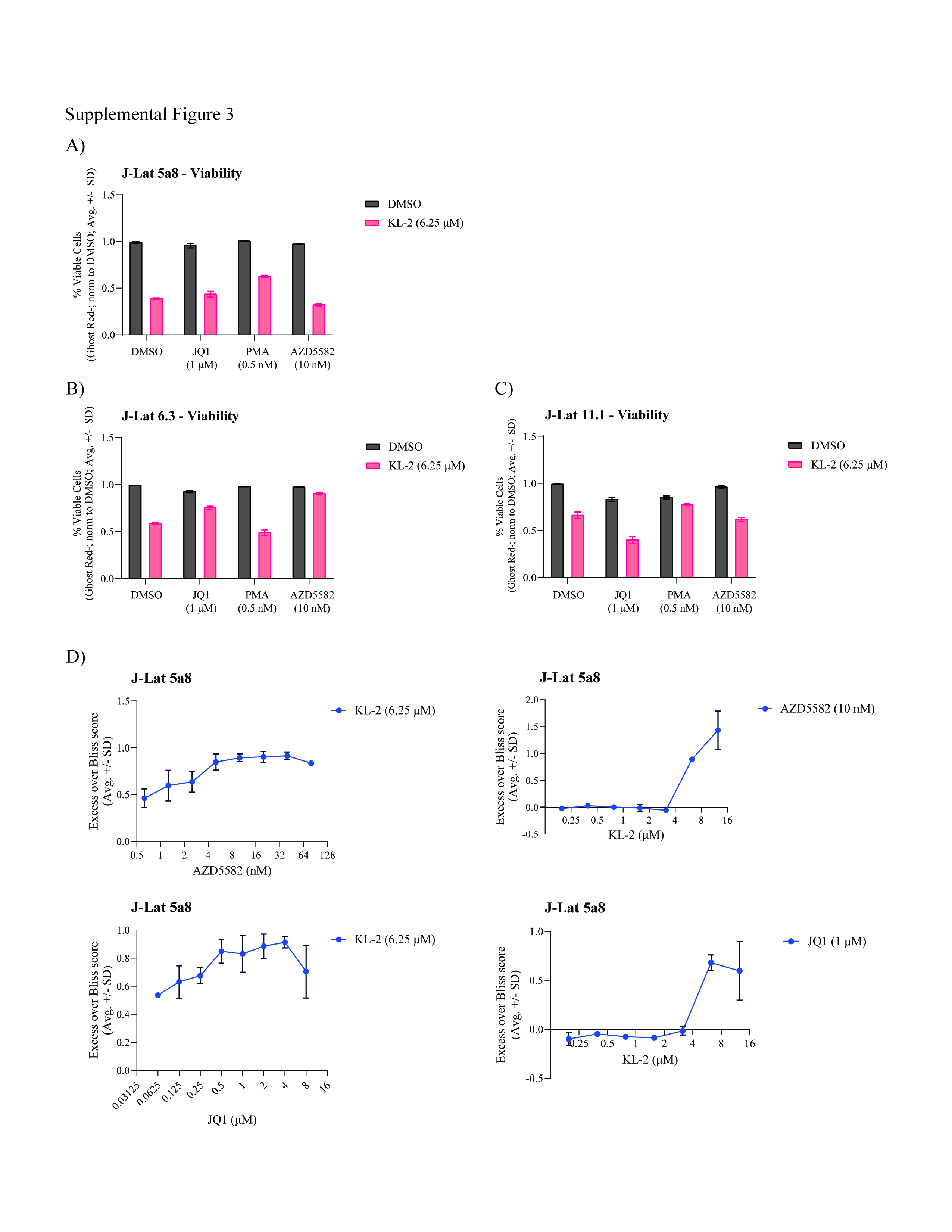

Supplement: S3 Fig — A) Percent viable J-Lat 5A8 cells (normalized to the DMSO-treated control) 48 hours after treatment with the indicated LRAs in the presence and absence of 6.25 μM KL-2 as measured by amine dye staining and flow cytometry. Each bar represents the average ± standard deviation of technical triplicates. The same data are shown for B) J-Lat 6.3 cells and C) J-Lat 11.1 cells. D) Excess over Bliss scores for latency reactivation in J-Lat 5A8 cells treated with combinations of KL-2 and AZD5582 (top) or JQ1 (bottom). Cells were treated in biological duplicates with the mean excess over Bliss score +/- standard deviation between replicates shown. (TIF) [file ppat.1012083.s003.tif]

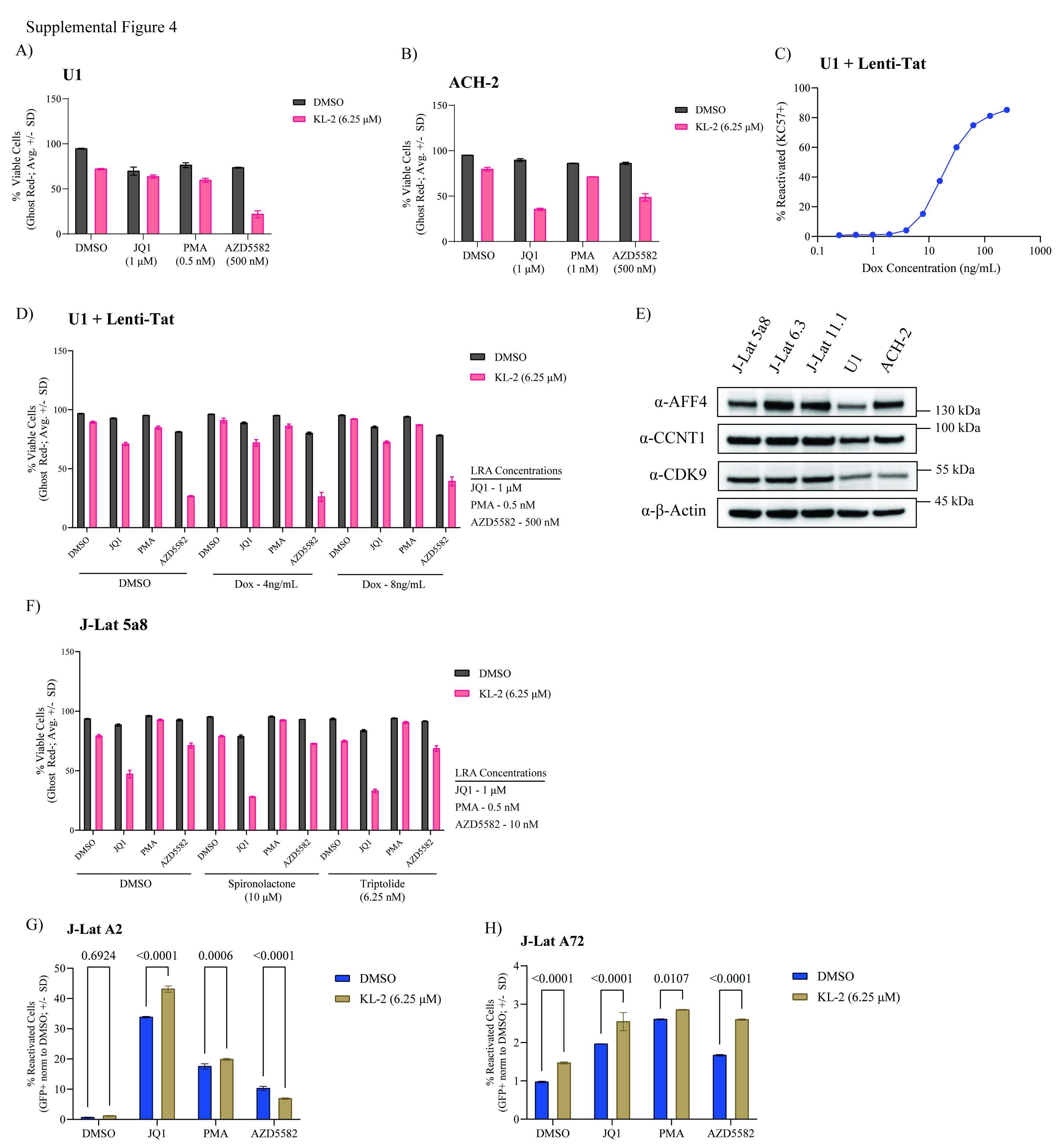

Supplement: S4 Fig — A) Percent viable U1 cells 48 hours after treatment with the indicated LRAs in the presence and absence of 6.25 μM KL-2 as measured by amine dye staining and flow cytometry. Each bar represents the average ± standard deviation of technical triplicates. B) Percent viable ACH-2 cells 48 hours after treatment with the indicated LRAs in the presence and absence of 6.25 μM KL-2 as measured by amine dye staining and flow cytometry. Each bar represents the average ± standard deviation of technical triplicates. C) Percent reactivated (KC57-FITC+) Lenti-Tat U1 cells after 48 hours of treatment with increasing concentrations of doxycycline. D) Percent viable Lenti-Tat U1 cells 48 hours after treatment with the indicated LRAs in the presence and absence of 6.25 μM KL-2 and differing amounts of doxycycline as measured by amine dye staining and flow cytometry. Each bar represents the average ± standard deviation of technical triplicates. E) Immunoblotting of whole cell lysates from the indicate cell lines using primary antibodies against AFF4, CCNT1, CDK9, and β-Actin. F) Percent viable J-Lat 5A8 cells 48 hours after treatment with the indicated LRAs in the presence and absence of 6.25 μM KL-2 and the Tat inhibitors Spironolactone and Triptolide as measured by amine dye staining and flow cytometry. Each bar represents the average ± standard deviation of technical triplicates. G) Percent reactivated (GFP+) J-Lat A2 cells (normalized to the DMSO control) after 48 hours of treatment with increasing concentrations of KL-2. Data represent the average ± standard deviation of technical triplicates; statistics were calculated relative to the DMSO control by two-way ANOVA and Sidak’s Multiple Comparison test. H) Percent reactivated (GFP+) J-Lat A72 cells (normalized to the DMSO control) after 48 hours of treatment with increasing concentrations of KL-2. Data represent the average ± standard deviation of technical triplicates; statistics were calculated relative to the DMSO control by two [file ppat.1012083.s004.tif]
